# The phylogenetic significance of the morphology of the syrinx, hyoid and larynx, of the southern cassowary, *Casuarius casuarius* (Aves, Palaeognathae)

**DOI:** 10.1186/s12862-019-1544-7

**Published:** 2019-12-27

**Authors:** Phoebe L. McInerney, Michael S. Y. Lee, Alice M. Clement, Trevor H. Worthy

**Affiliations:** 1 College of Science and Engineering, Flinders University, Adelaide, SA Australia; 2 South Australian Museum, Adelaide, SA Australia

**Keywords:** Palaeognathae, Cassowary, Syrinx, Hyoid, Larynx, Morphology, Phylogenetics, Optimisation

## Abstract

**Background:**

Palaeognathae is a basal clade within Aves and include the large and flightless ratites and the smaller, volant tinamous. Although much research has been conducted on various aspects of palaeognath morphology, ecology, and evolutionary history, there are still areas which require investigation. This study aimed to fill gaps in our knowledge of the Southern Cassowary, *Casuarius casuarius*, for which information on the skeletal systems of the syrinx, hyoid and larynx is lacking - despite these structures having been recognised as performing key functional roles associated with vocalisation, respiration and feeding. Previous research into the syrinx and hyoid have also indicated these structures to be valuable for determining evolutionary relationships among neognath taxa, and thus suggest they would also be informative for palaeognath phylogenetic analyses, which still exhibits strong conflict between morphological and molecular trees.

**Results:**

The morphology of the syrinx, hyoid and larynx of *C. casuarius* is described from CT scans. The syrinx is of the simple tracheo-bronchial syrinx type, lacking specialised elements such as the pessulus; the hyoid is relatively short with longer ceratobranchials compared to epibranchials; and the larynx is comprised of entirely cartilaginous, standard avian anatomical elements including a concave, basin-like cricoid and fused cricoid wings. As in the larynx, both the syrinx and hyoid lack ossification and all three structures were most similar to *Dromaius.* We documented substantial variation across palaeognaths in the skeletal character states of the syrinx, hyoid, and larynx, using both the literature and novel observations (e.g. of *C. casuarius*). Notably, new synapomorphies linking Dinornithiformes and Tinamidae are identified, consistent with the molecular evidence for this clade. These shared morphological character traits include the ossification of the cricoid and arytenoid cartilages, and an additional cranial character, the articulation between the maxillary process of the nasal and the maxilla.

**Conclusion:**

Syrinx, hyoid and larynx characters of palaeognaths display greater concordance with molecular trees than do other morphological traits. These structures might therefore be less prone to homoplasy related to flightlessness and gigantism, compared to typical morphological traits emphasised in previous phylogenetic studies.

## Background

Palaeognathae is one of two primary avian clades. Considered to have diverged during the middle Cretaceous [1–3], Palaeognathae comprises the volant tinamous (Tinamidae) (South America) and the flightless, cursorial ratites such as the Australian emu (*Dromaius*) and the extinct New Zealand Moa (Dinornithiformes) ([2, 4], p., 272, [5–7]). The large, cursorial southern cassowary, *Casuarius casuarius,* is one of three cassowary species which along with the Australian emu, forms the family Casuariidae, a group nested within Palaeognathae [8]. The southern cassowary is endemic to the tropical rainforests of New Guinea and Australia [8, 9]. It has a solitary nature [10] and a preference for dense forested habitats [11], hence relatively little is known about cassowary ecology in comparison to its extant relatives [12]. These gaps in knowledge extend to the phenotype: poorly studied structures in the cassowary include the syrinx, hyoid and larynx, despite morphological and comparative analyses of these structures in other palaeognaths, and their importance for primary biological functions and potentially phylogenetic inferences.

Birds primarily vocalise through the movement and manipulation of syringeal elements within the syrinx, and so vocal output is constrained by the mechanical design of this organ within the vocal tract [13–16]. The syrinx has been described for numerous taxa, revealing substantial morphological variation, often reflecting differential vocalisation demands [17]. Due to the simplicity of palaeognath syrinx structures, nineteenth century zoologists claimed ratites lacked syringeal characteristics, and therefore, had no syringeal organ. However, in the late 1800s this assumption was challenged, with Forbes [18] proving the presence of several syringeal structures, such the tympaniform membranes. Undoubtedly though, palaeognaths have structurally very simple syringes in comparison to those of many neognaths ([19] p. 123), leading to suggestions that they represent either an example of evolutionary degeneration and/or retention of an unspecialised and primitive form ([18, 19], p., 123, [20], pp., 60-65). The apparent simplicity of the organ may have contributed to the cassowary syrinx receiving only superficial study [18–21]; an absence of detailed comparative studies with other palaeognath taxa is also notable.

In Aves, the laryngeal and hyoid apparatus form the floor of the oropharyngeal cavity ([22, 23], p., 50), sitting directly beneath the mandible ([24], p., 386). As individual structures comprising their own skeleton and complex musculature, both work independently and in synchrony to facilitate respiration and feeding ([22, 23], p., 50, [25, 26], p., 69). The hyoid apparatus forms an essential structural element within the upper digestive tract, supporting and controlling the lingual corpus, the tongue ([22, 23], p., 51, [26], p., 77, [27, 28]). The larynx attaches to the dorsal aspect of the hyoid corpus, and acts as the gateway into the trachea, a barrier to foreign bodies entering the respiratory tract during swallowing ([23], p., 50, [25, 26], p., 77).

The literature on the syrinx, hyoid apparatus and larynx demonstrates that these organs not only play key functional roles but exhibit considerable variation in morphology among taxa [25, 29]. Thus, a descriptive analysis for individual taxa is essential to the development of a comprehensive understanding of the organs [25]. The syrinx and hyoid have also proven valuable in phylogenetic inference among various neognath clades, including the suboscine family Rhinocryptidae [30], providing novel phylogenetic characters, informing relationships and assisting in the classification of taxa by morphologists and systematists [6, 30–35]. Therefore, it is likely analysis of these structures could be similarly beneficial within palaeognath phylogenetics.

The hyoid apparatus and syrinx of the cassowary have previously been described, primarily during the late 1800s and 1900s (for example [18, 19, 21, 36, 37]). However, these descriptions are brief and often lack context, partly due to authors not having the technology now available. Furthermore, there is no description of the cassowary larynx, despite it being described for other palaeognaths. Clearly, further morphological analysis of these structures is desirable and may prove phylogenetically important [6, 31–34, 38], as found for aforementioned neognath clade, Rhinocryptidae [30]. Phylogenetic analyses of palaeognaths have revealed discordant topologies between morphological and molecular data, thought to be a result of convergent morphological traits, with genomic data largely driving the current consensus [1, 3, 5, 7, 39]. Research has identified few morphological characters in support of relationships found in molecular-based analyses; most are cranial characters, not associated with structures related to cursorality and flight which might be susceptible to convergence [2, 39]. This suggests analysis of overlooked structures unrelated to locomotion, such as the syrinx, hyoid apparatus and larynx, may also retrieve evolutionary patterns more similar to that of the molecular evolutionary tree for palaeognath taxa. Thus, we herein present new anatomical observations and comparative analyses of the skeletal structures of the syrinx, hyoid, and larynx (SHL) skeletal structures of the Southern Cassowary, *Casuarius casuarius,* and identify morphological characters within these structural systems which contribute to improved resolution of phylogenetic relationships.

## Results

### Syrinx

**Fig. 1:**
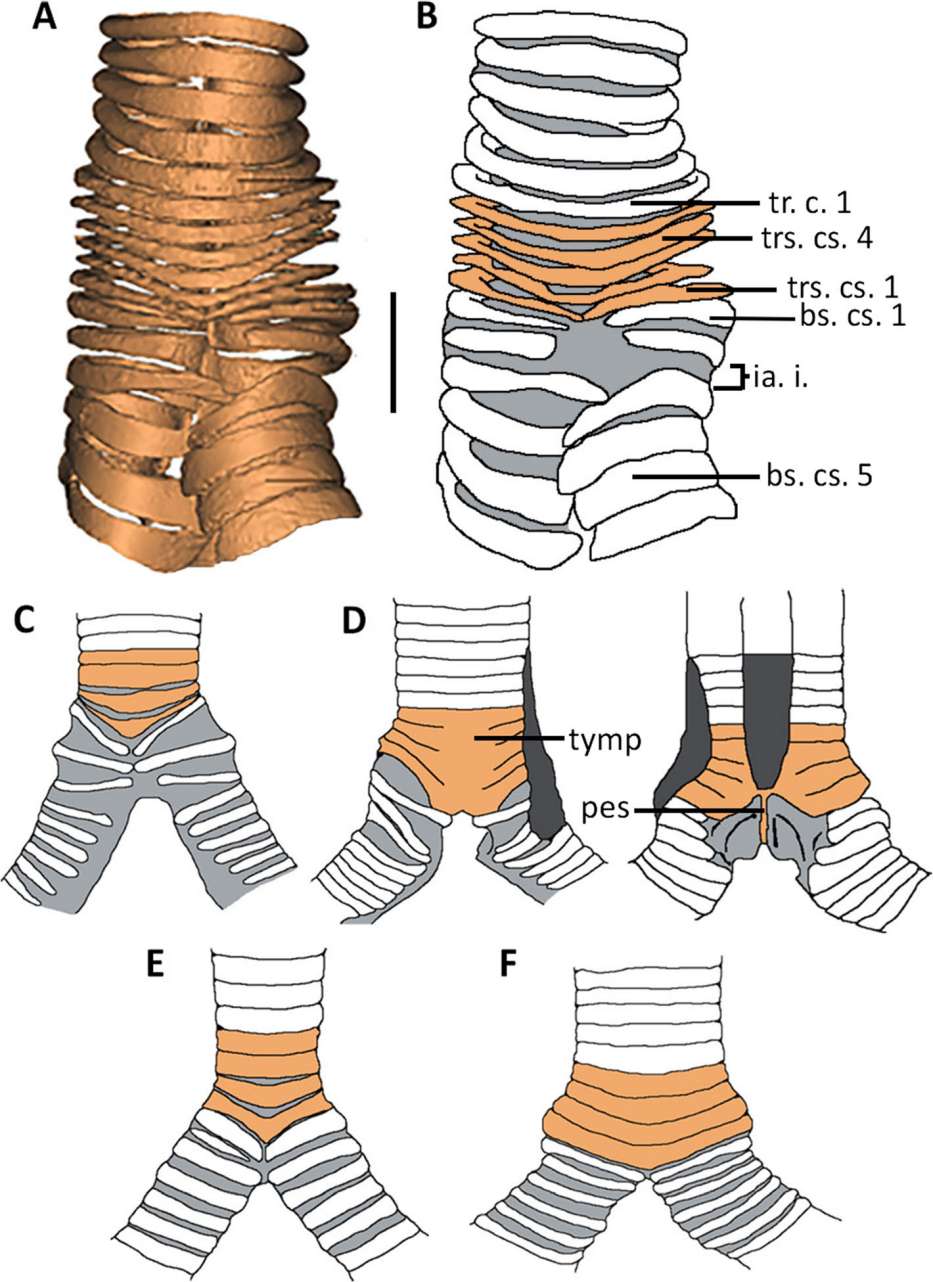
Palaeognath syringeal elements with tracheosyringeal cartilages differentiated by brown shading (**b**-**e**), interannular tissues are shaded in grey. **a**-**b** Cassowary, *Casuarius casuarius*, FUR180; ventral, scale bar = 10mm. **c** Tinamou, *Nothura darwinii*, adapted from Garitano-Zavala ([40], fig. 1C); ventral. **d** Rhea, *Rhea americana*, adapted from Forbes ([18], fig. 7, 8); ventral and dorsal. **e** Kiwi, *Apteryx mantelli*, adapted from Forbes ([18], fig. 3), ventral. **f** Ostrich, *Struthio camelus*, adapted from Forbes ([18], fig. 1); ventral. Abbreviations: bs.cs: bronchosyringeal cartilages, ia.i: interannular interval, tr.c: tracheal cartilages, trs.cs: tracheosyringeal cartilages. **c**-**f** scaled to same size approximately

Among Aves, three syringeal types are recognised and differentiated by topographical position of the elements contributing to the syrinx ([17, 19], p., 107, [20], p., 61). The tracheo-bronchial syrinx is the most common form, located at the bifurcation of the trachea into the two bronchial tubes, and includes both tracheal and bronchial elements ([19], p., 109, [20], p., 61). The tracheal syrinx sits within the tracheal tube, whereas the bronchial syrinx develops as two semi-syringes along each bronchi ([19], p. 107–109, [20], p. 61). As the syrinx of the cassowary (FUR180) structurally conforms to the tracheo-bronchial syrinx type (Fig. 1a-b), as do those of all palaeognaths ([20] p. 61), only the morphology of this type will be discussed. The basic structure of this type of syrinx consists of modifications to the caudal end of the trachea and the cranial portions of the two bronchi to form the tracheosyringeal and bronchosyringeal cartilages respectively ([19], p., 107, [35]). Other morphological features often include the fusion of the tracheosyringeal cartilages to form a rigid box-like structure termed the tympanum, and a pessulus, a bridge traversing the base of the tracheal tube in the dorso-ventral plane ([19], p., 110, [20], p., 61, [40]).

The syrinx of FUR180 is simple and comparable in form with others previously described for the cassowary, such as the adult male assessed by King ([19] p. 124), indicating the absence of sexual dimorphism in this organ. Of the palaeognaths, and all birds, the ostrich has been considered to have one of the simplest syringes, with which it can produce a limited repertoire of sounds (Fig. 1f) [41]. However, the ostrich syrinx does have a pessuliform process and potentially also has a tympanum [18, 42], indicating a more derived state than in other palaeognaths including the cassowary. The syrinx of the rhea (Fig. 1d) is the most complex, due to the presence of specialised anatomical structures, including fully developed intrinsic musculature, absent in most other palaeognaths [18, 21, 43].

#### Cart. Tracheosyringeales

There are five tracheosyringeal cartilages associated with the cassowary syrinx, distinct from preceding true tracheal cartilages as they are thinner ventrally and laterally. Dorsally all are incomplete along the midline, with the extremities bending medially towards the centre of the syrinx, a result of tracheosyringeal membranes contracting post-death [21]. Forbes [18] and Pycraft [21] noted the presence of these imperfect tracheosyringeal cartilages, proposing the cranio-caudal space formed between the cartilage extremities is occupied by transversely running fibrous and elastic tissues [21] later termed the tracheosyringeal membranes (*mem. tracheosyringealis*) ([19] p. 128). Dorsally incomplete cartilages are present in the ostrich, moa, kiwi and emu, although are lacking in the rhea and tinamou. The number of incomplete cartilages varies depending on the species, although no other species approaches having nine, the number present in the cassowary specimen. Three have been noted for the moa [44] and emu [18, 21], and two for the ostrich [18]. The number present in the kiwi is dependent on the species; *Apteryx australis* has three compared to the single incomplete cartilage in *A. mantelli* [18].

All five tracheosyringeal cartilages angle caudally along the medial line of the ventral side of the cassowary syrinx, with the degree of the angle increasing caudally with each cartilage, paired with a cranio-caudal increase of width. These features are common in palaeognaths, although variable in some taxa such as the ostrich and kiwi, which develop this character on the dorsal side of the syrinx. Ventral modification to the most caudal tracheosyringeal cartilages in the moa differentiates this taxon from others: the cartilage lengthens cranio-caudally at the most caudal point of the V, from which a caudo-medially directed projection extends [44]. Oliver [44] interpreted this keel syringeal ring to likely be the ventral attachment point of a pessulus.

Noted as a common feature among casuariids (*Dromaius* and *Casuarius*) by Pycraft [21], the cassowary syrinx shows poor transitional definition between bronchosyringeal and tracheosyringeal cartilages. The most caudal tracheosyringeal cartilage (trs. cs. 1) closely reflects the structure of the first bronchosyringeal cartilage with cartilages differentiated only by partial fusion of the ventral extremities present in the former, maintaining a single element structure. Tinamous also display a gradual transition between cartilage types [40], although greater transitional definition is noted in the ostrich, kiwi and rhea. The ostrich trachea increases in diameter in the few cartilages preceding tracheal bifurcation; the following bronchosyringeal cartilages are much narrower craniocaudally [18, 42]. Alternatively, distinction between the two cartilage types in kiwi is formed from a widening of the bronchosyringeal cartilages after tracheal bifurcation [18, 21]. The transitional definition in the rhea is unique among palaeognaths with the fusion of tracheosyringeal cartilages forming a tympanum just cranial to tracheal bifurcation [18, 21, 43].

#### Pessulus

No pessulus is present in the cassowary specimen FUR180, with the left and right medial tympaniform membranes fusing transversely along the dorso-ventral plane, at the level of tracheal bifurcation. As expected, no intrinsic musculature was found in the μCT-generated model as expected given its absence in specimens described by Forbes [18] and Pycraft [21]. Similarly, the tinamous [40], kiwi [18, 21] and emu [18] also lack a pessulus. In the rhea, this structure is present; the pessulus links the caudo-medial point of the dorsal and ventral sides of the tympanum as a narrow osseous bridge [18]. Ossification has been recorded for rhea, despite the structure being primarily cartilaginous, suggesting that increased ossification occurs later in ontogenetic staged in males. Based on a described structure by Owen [45], Oliver [44] reported that moa also develop an ossified pessulus forming a partial bridge across the ring and likely completed by cartilage. Observations of moa syringeal elements in the NMNZ collection (see Additional file 1: SI 1) support the formation of a partial pessulus with identified keeled syringeal rings. Early observations of the ostrich syrinx found the third tracheosyringeal cartilage to contain a short caudal projection, medially on the ventral border [18]. This is considered a pessuliform process; not a true pessulus due to it not traversing the ventrodorsal width of the trachea. Yildiz and colleagues [42] noted the presence of a double-folded structure formed of connective tissue, suggesting this may act similarly to a true pessulus, providing support for the medial tympaniform membranes.

#### Tympanum

FUR180 has complete lack of fusion between tracheosyringeal cartilages indicating a tympanum is absent in cassowaries. No evidence was found to support Forbes’ [18] claim for the presence of an ‘expanded’ tympanum, and it is unlikely that incomplete and unfused cartilages such as we observe could function similarly to a true tympanum.

Among all palaeognath taxa, a tympanum has only been described in the ostrich, rhea, and moa; this structure is absent in tinamous ([40], data herein). However, among these taxa in which a tympanum has been reported, only in rhea has the presence been confirmed with complete dorsal and ventral fusion of four to six tracheosyringeal cartilages [18, 21, 43]. Fusion between tracheosyringeal elements has been described in some ostrich specimens, with the tympanum comprising three tracheal cartilages, although Yildiz et al. [42] found the cartilages only appeared to be fused through the presence of ligamentum annulare. Oliver [44] also provides a description pertaining to the presence of a possible tympanum in moas, located cranial to the tracheosyringeal cartilages ([46] p. 107). We searched numerous moa specimens and provide in the Additional file 1: SI 1 a list by species and presence of tracheal rings, noting the type of rings present including the unique syringeal keeled ring that represents an incomplete ossified pessulus. The tympanum described by Oliver ([44], Fig 26) was not found among any tracheal ring sets present in any moa taxa (Additional file 1: SI 1). We consider that given the keeled syringeal ring is often present, then there was no tympanum in moa, given such would incorporate this ring with more than one other, making a relatively robust ossified element more likely to survive and be found with a skeleton than any individual ring.

#### Cart. Bronchosyringeales

The cassowary bronchi are asymmetrical, with the left bronchus larger in diameter. Also contributing to the asymmetry, the ventral extremity of the third bronchosyringeal cartilage on the left bronchi is angled ventro-medially and expands caudally, both characters are absent on the right bronchi, although both sides display an increase in cartilage length from preceding cartilages. The medial extremities of the bronchosyringeal cartilages overlap dorsally and ventrally, indicating a potential for expansion of interannular intervals during use. Interannular intervals (spaces) between bronchosyringeal cartilages remain relatively uniform for the length of the specimen, with the absence of large, paired intervals indicating lateral tympaniform membranes do not develop. The kiwi similarly possesses uniform intervals [18], whereas in the ostrich, ventral intervals narrow, and cranial intervals widen [18, 42]. The tinamou possesses two wide interannular intervals between the first to the fourth bronchosyringeal cartilages [40]. The first two bronchosyringeal intervals indicate the presence of the lateral tympaniform membrane, also noted for the rhea.

### Hyoid

**Fig. 2:**
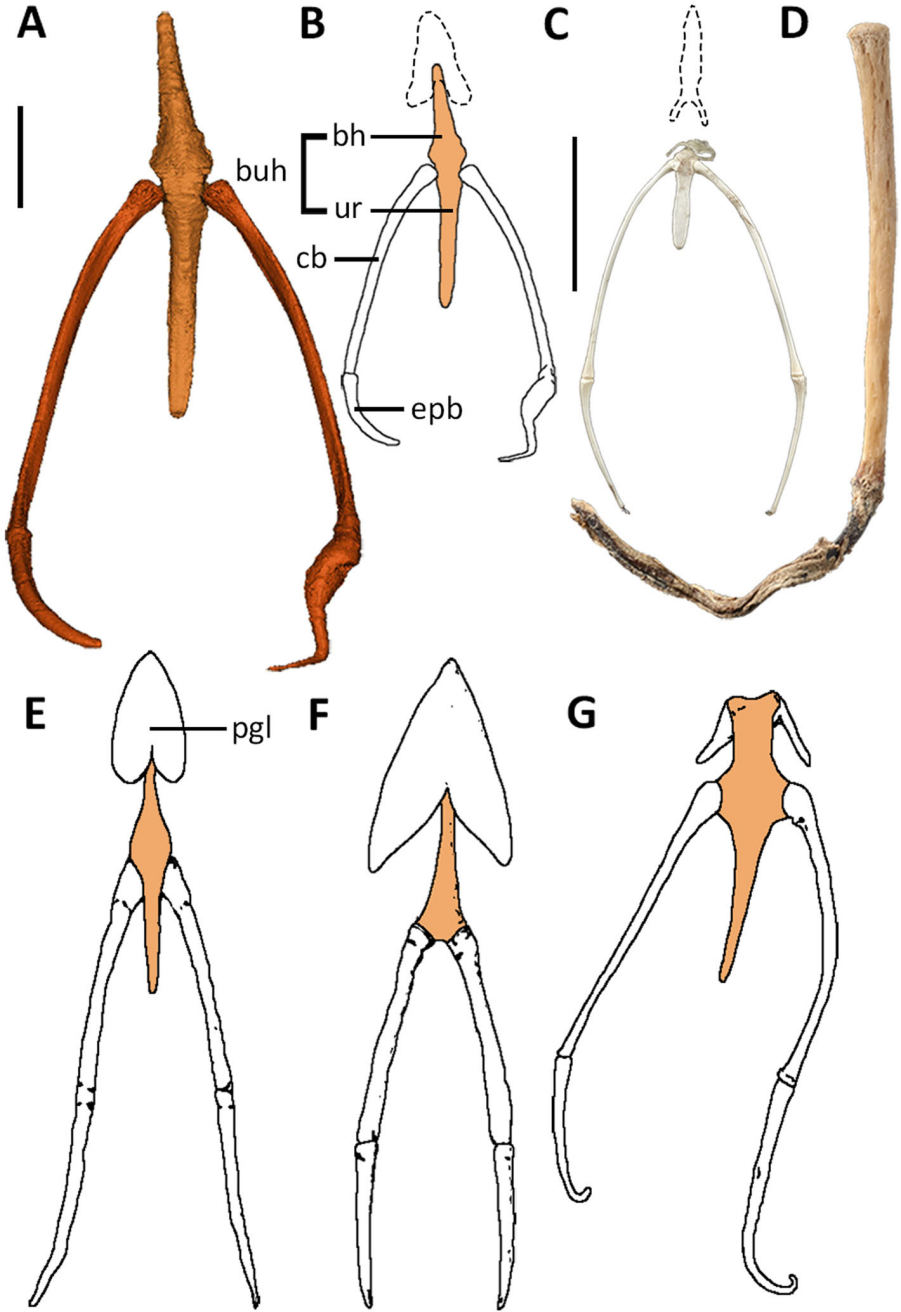
Palaeognath hyoid elements. **a**-**b** Cassowary, *Casuarius casuarius*, FUR180; dorsal view, A- scale bar = 10mm. **c** Tinamou, *Nothoprocta perdicaria*, NMNZ S. 22983; dorsal view, scale bar = 10mm. **d** Moa, *Megalapteryx*, NMNZ S. 400; dorso-lateral view, scale bar = 10mm. **e** Emu, *Dromaius novaehollandiae,* adapted from Parker ([36], plate XII); dorsal view. **f** Rhea, *Rhea americana,* adapted from Parker ([36], plate X); dorsal view. **g** Ostrich, *Struthio camelus*, adapted from Soley *et al.* ([22], fig. 3); dorsal view. Abbreviations: bh: basihyal, buh, basiurohyal, cb: ceratobranchial, epb, epibranchial, ur: urohyal. Dotted lines indicate potential paraglossal shape for the cassowary (**b**) and tinamou (**c**), derived from the hyoids of closely related species figured in Parker ([36], see plates 14 and 15)

All typical hyoid skeletal components are present within the hyoid apparatus of FUR180 (Fig. 2a-b). The basihyal, urohyal, and a paraglossal are cartilaginous, with only the ceratobranchials ossified. Ossification of ceratobranchials is common to all Aves, while the extent to which other elements ossify varies by lineage ([22, 24], p., 367, [46], p., 110, [47, 48]).

#### Basiurohyal

The joint between the basihyal and urohyal is indiscernible, with complete fusion of the two cartilaginous skeletal elements forming the basiurohyal (Fig. 2b). This character is common among palaeognaths; the only exception is the rhea in which the urohyal is lost (Fig. 2f) ([24], p., 369, [36, 49]). Rostrally, the basiurohyal curves dorsally, preceding a slight ventral arch centrally along the corpus, with the caudal point terminating with a minor inwards hook towards the laryngeal cricoid cartilage. The basiurohyal in the cassowary and emu have rounded tips [27], compared to that of the ostrich (Fig. 2g) which tapers caudally to a pointed tip, and rostrally terminates in two bulbous projections divided by a shallow notch [22, 27, 36]. The basihyal of the rhea is more cylindrical than in other palaeognaths although it also terminates in a rounded rostral tip ([24], p., 366, [49]).

Despite the basiurohyal being completely unossified in the cassowary, ossification of basiurohyal elements does occur in the kiwi [21] and rhea [49]. A partially ossified basiurohyal was also identified in a single moa specimen (*Megalapteryx*, specimen no. NMNZ S.400). The identification of an ossified moa basiurohyal shows that partial ossification may exist in at least the basal moa genus *Megalapteryx* though a lack of these elements in the moa fossil record limit our ability to determine whether this is an incident caused by the ontogenetic age of the specimen. We also identified a basiurohyal in which the urohyal and caudal portion of the basihyal were ossified in a tinamou (Fig. 2c) (*Nothoprocta perthicaria*, NMNZ S.22983). In an earlier study, Li et al. [48] found that despite midline ossification being a key component of the hyoid apparatus in neognath birds, the degree of ossification in palaeognaths varies and is often incomplete when present. This is correct for most palaeognaths, however even our limited observation of tinamou and moa specimens show complete ossification can be present. Therefore, further investigations across a wider sample of taxa is required to fully assess this variability.

#### Ceratobranchiale and epibranchiale

The basiurohyal articulates mid-way along its lateral edges with the ceratobranchials. The bulbous, disk-like proximal ends of the ceratobranchials sit low in the concave basiurohyal sockets; the articular surface of the sockets is larger than that of the proximal ceratobranchial end, indicating an allowance for considerable movement within the joint. The ceratobranchials are elongate with one third of their length extending past the caudal point of the basiurohyal. The shaft curves dorsally towards the ceratobranchial-epibranchial joint, from which the shorter cartilaginous epibranchials extend caudo-dorsally. In our specimen the right epibranchial is deformed with a large, sharp medial bend and increased tissue mass, contrasting with the smooth curve of the left epibranchial. The lack of symmetry, as well as no previous mention of such deformity within the literature for the cassowary or any other palaeognath taxa, indicates a pathology. The shape would impede the functionality of the epibranchial, including movement in and out of the hyoid sheath.

Palaeognath ceratobranchials are often cylindrical in shape, although can be slightly flattened as in the rhea [36]. Ostrich epibranchials are elongate ([24] p. 371) compared to most other palaeognaths, which have short epibranchials relative to the ceratobranchials ([24] p. 367). In the tinamou, both epibranchials and ceratobranchials display increased elongation [36] compared to other taxa. We found the tinamou to have ossified epibranchials, differing from other palaeognaths including its closest relative, the moa, in which the epibranchials do not ossify (Fig. 2d).

#### Paraglossum

The cranial portion of the hyoid skeleton attaches to the paraglossal; both structures are encased by soft tissue, connecting the paraglossal to the back of the tongue body. The cassowary paraglossal is a single un-ossified element seemingly with a rounded triangular shape, as suggested by Parker [36]. The paraglossal of the emu (Fig. 2e) is similarly tear-drop shaped, although the caudal edge may be rounded or scalloped [27, 36, 50]. The shape of the paraglossal in the rhea reflects the more triangular shape of the tongue, although it is smaller and with an oval opening dorsal on the palate [49]. The tinamou paraglossal is much narrower in width than other species, with scalloped margins and two caudally directed projections, one from each caudo-lateral corner ([24], p., 372, [36]). Unique among palaeognaths, the ostrich paraglossal is divided into two narrow, caudo-laterally directed individual paraglossia, situated ventro-laterally in the tongue body ([24], p., 371, [27, 51]). As the ostrich is phylogenetically basal among palaeognaths, this paired state could potentially be plesiomorphic, the rhea then shows partial fusion and the other palaeognaths, complete fusion.

#### Lingual corpus

When compared to the tongues of other avian taxa, palaeognath tongues are significantly shorter relative to the mandible; they have thus been described as vestigial organs, rudimentary in morphology [28, 51]. The cassowary tongue is no exception, reflecting the limited role played by the tongue during the ‘catch and throw’ feeding method, a method utilising obligate inertial feeding in which the tongue is unrequired ([23], pp., 53, and, 74, [52, 53]).

As in the rhea, emu, and ostrich ([24] pp. 366-371, [28, 49, 50, 54]), the cassowary tongue is cranio-caudally flattened and triangular. Only the tongue of the kiwi varies significantly, being thinner and less triangular than in other taxa [55], reflecting the shape of the long and narrow bill. The narrow tongue of the kiwi is likely a result of dietary specialisation; the elongated bill is required for detecting buried or submerged prey using vibration-sensitive mechanoreceptors [56, 57]. The cassowary tongue corpus has a smooth and rounded rostral apex as in tinamous and ostriches, although varying from the pointed tip of the rhea tongue ([24], p., 372, [49]). The caudal and rostral edges of the tongue are concave, although the rostral notch is more prevalent in the rhea, ostrich, and tinamou, than the cassowary and emu ([24], p., 372, [49, 58]).

Numerous lingual papillae, arranged asymmetrically, line the sides of the cassowary tongue, increasing in length and width caudally towards the tongue base. Although the emu displays analogous structures, these are not present in all palaeognaths. The tongue of the rhea lacks lateral papillae although the caudo-lateral corners project caudally [28, 49, 50, 58]. Both absence and presence of papillae have been noted for the ostrich ([24], p., 371, [27]), and papillae are completely absent in tinamous ([24] p. 372). Only the emu tongue is considered to have caudal papillae, although poorly defined and rudimentary when compared to those directed laterally [29, 50]. Lingual papillae are absent on the dorsal surfaces of all palaeognathous tongues [28].

### Larynx

**Fig. 3:**
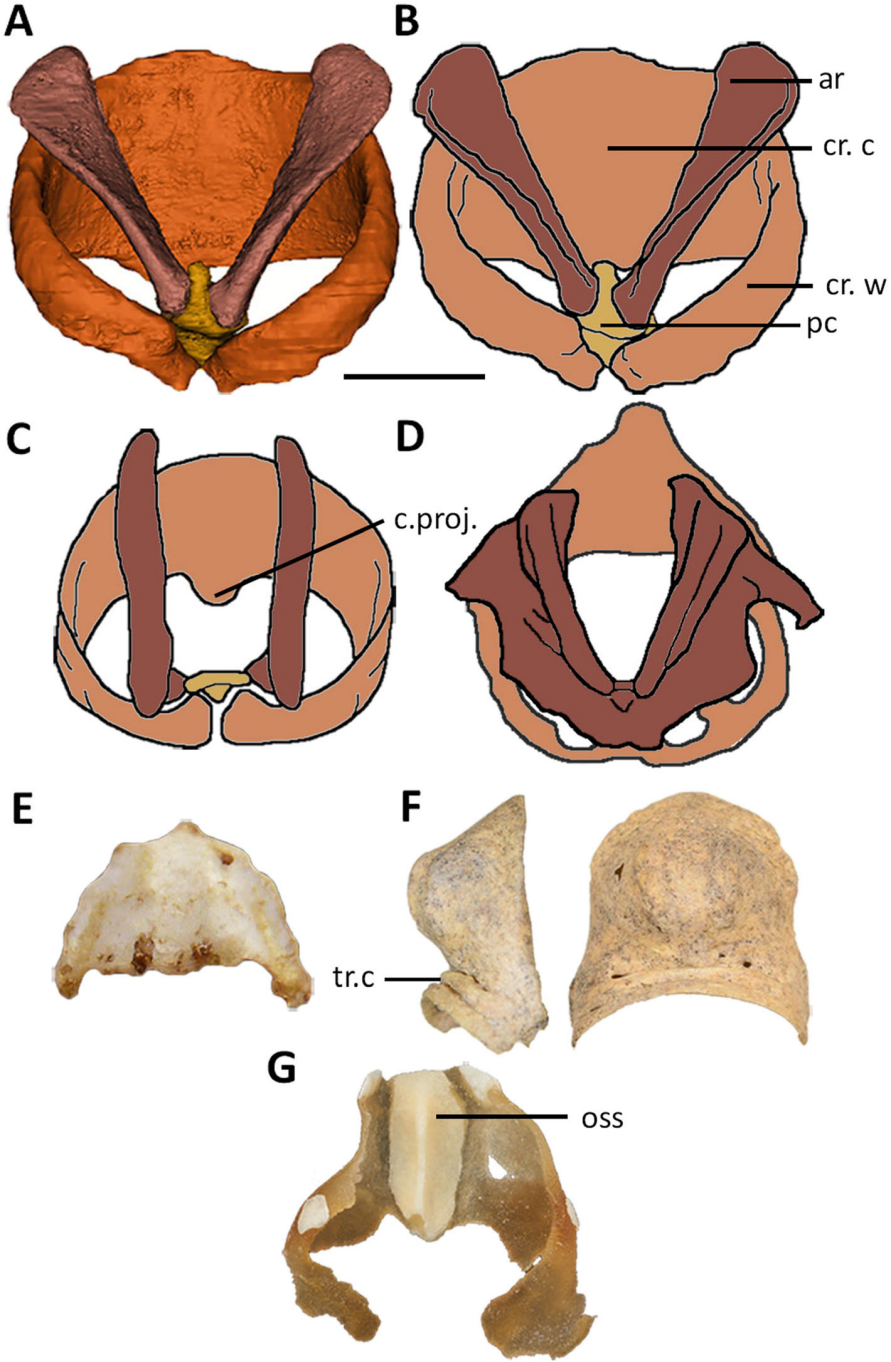
Palaeognath laryngeal elements. **a**-**b**, Cassowary, *Casuarius casuarius*, FUR180; dorsal view, scale bar = 10mm. **c** Rhea, *Rhea americana,* adapted from Crole and Soley ([49], fig. 7); dorsal view. **d** Ostrich, *Struthio camelus*, adapted from Tadjalli ([47], fig. 9a, b); dorsal view. **e** Tinamou, *Nothura maculosa*, MMC321; ventral view. **f** Moa, *Euryapteryx curtus*, NMNZ S. 44757; lateral and ventral views. **g** Kiwi, *Apteryx rowi*, NMNZ OR.27243A; dorsal view. Abbreviations: ar: arytenoids, cr.c: cricoid cartilage, cr.w: cricoid wings, oss: ossification, pc: procricoid, tr.c: tracheal cartilages

The larynx of the cassowary FUR180 (Fig. 3a-b) has a standard avian anatomy, composed of the cartilaginous skeletal elements, the cricoid, procricoid, and paired arytenoid cartilages. The larynx in FUR180 is entirely cartilaginous as in the emu, some palaeognaths (ostrich [47], rhea [49] and kiwi) have poorly and variably defined ossification centres in the corpus, with partial ossification in the rhea [49] and kiwi (personal observation, Fig. 3) likely dependent on ontogenetic stage. Only tinamous and moa have a strongly ossified cricoid corpus where the entire corpus is well ossified and has well defined margins resulting in a distinctive cricoid bone. This character is thus identified as a synapomorphy supporting the molecular-based pairing of this clade. To date, morphological characters supporting the molecular identification of this clade [3, 7, 39] have remained elusive and thus, these findings are significant and provide phylogenetically informative data.

#### Cricoid cartilage

The cassowary cricoid in FUR180 is characterised by a concave plate- or basin-like corpus, with a smooth dorsal and ventral surface. Neither the cassowary, emu, rhea, nor ostrich develop a median ridge traversing the dorsal (inner) surface of the main ventral plate/bowl of the cricoid ([26], p., 73, [47, 49]). This crista is present in the kiwi ([26] p. 72), moa and tinamous, projecting dorsally into the laryngeal lumen. Some moa species have two ridges of varying heights ([46], p., 106, [59]), similar to the cricoid of *N. maculosa* which has two slightly raised ridges. The inverse of these ridges are recognisable on the ventral surface of the cricoid in both taxa (Fig. 3e, f). The lateral margins of the moa cricoid are smooth (Fig. 3f); this is also noted within the tinamou species *E. elegans*, although varying from the scalloped margins of the cricoid in *N. maculosa* (Fig. 3e). A small medially located cartilaginous caudal projection has been observed in the rhea (Fig. 3c). This projection is often fused with tracheal cartilages and, in some species, is situated between two smaller, caudo-medially directed extensions [49]. Many moa genera, including *Dinornis* and *Pachyornis*, also develop these features [44, 59]. Caudal projections are absent in the cassowary. The emu and ostrich develop a rostral process ([22, 26], p., 73, [47]) which ossifies in the ostrich, acting as an attachment point for the cartilaginous basiurohyal.

Cartilaginous cricoid ‘wings’ extend seamlessly dorsocaudally from the lateral margins of the cricoid cartilage in the cassowary, ostrich, emu, and rhea. As no moa cricoid wings have been identified, despite the collection of multiple cricoid bones from fossil deposits, it is likely this element was also cartilaginous in moa. Ossification of the cricoid in moa ([44, 46], p., 106) and tinamou (observations herein), indicates that the wings fused to the lateral borders of the cricoid were cartilaginous. This hypothesis is supported by images of tinamou cricoids (*Nothura maculosa* MMC 321*, Eudromia elegans* MMC 350, *Rhynchotus rufescens* MMC 358) (Marcos Cenizo, Museo de Historia Natural de La Pampa, see Additional file 2: SI 2). In all palaeognath taxa, the cricoid wings narrow dorsally and are directed caudally ([26], p., 73, [47, 49]). In the tinamou, rhea [49], emu and ostrich ([22, 26], p., 73, [47]), the cricoid wings join dorsally, completing the cricoid ring caudal to the procricoid. In the cassowary, the wings do not articulate dorsally; instead the procricoid and a cranial projection from the medial point on the dorsal side of the second tracheal cartilage insert between the wing extremities.

#### Cart. procricoidea

The cassowary procricoid cartilage is formed of a flattened rectangular corpus with a distal, cranially extending head, and a proximal, caudally directed tail. The tail is triangular, with the cricoid-procricoid joints on the flattened dorsal edge. The head is rounded cranially and flattened laterally, forming the dorso-medial walls of the concave basins which run ventrally along both sides of the procricoid. This concavity supports the arytenoids which extend rostro-laterally from the procricoid. Both the procricoid and paired crico-procricoid joints are seemingly caudo-medially supported by the cranial extension of the second tracheal cartilage. Dorsally, the emu procricoid is a simple rectangular shape ([26] p. 73), whereas the shape is wide and rounded rostrally in the ostrich. The ostrich procricoid also develops a ventro-caudally directed projection which extends between the cricoid wings [22]. The rhea procricoid is similar in shape although more angled, with a flattened rostral margin, and dorsally triangular caudal projection [49]. The dorso-cranial procricoid head, seen in the cassowary, is possibly absent in other palaeognaths, as is the cranial projection from the tracheal cartilages which sits below the procricoid. However, the literature provides no information on the procricoid for the tinamou, elephant bird or moa, with the descriptions for ostrich, rhea, and emu procricoids, brief and lacking detail.

#### Cart. arytenoidea

In the cassowary, the arytenoid corpus is cartilaginous, flat and elongated, extending laterally and ventrally to form two sides of a V-shape. The caudal end faces medially towards the opposing arytenoid and rests within the lateral procricoid joint concavities. Through the corpus and rostral projection, the flattened sides twist laterally to face dorsally. This arytenoid structure is shared with the emu, the closest relative of the cassowary although they vary with the lateral margins of the emu arytenoid converging rostrally into a cranio-medial point ([26] p. 73). The description of the arytenoid cartilage in the moa [44] suggests a similar structure, varying primarily in that the moa arytenoids are partly ossified. However, the few preserved moa arytenoids in the NMNZ show more extreme curvature along the element. In contrast, the arytenoids of the rhea are elongate paired bars with proximally directed projections extending from the caudo-dorsal aspect of the arytenoids for attachment to the procricoid [49]. The ostrich arytenoids are also elongated, although have thin cartilaginous plates extending from the lateral margins, unique among palaeognaths. The plates form two lateral, and one dorsal projection with smooth, rounded margins.

#### Glottis

The arytenoids are covered in a dense mucosa, which forms the glottis mound. The dorsal surface of the glottis mound is typically smooth in palaeognaths including the cassowary; the only exception being the tinamous ([26] p. 70). Prominent laryngeal papillae extend from a widened caudal margin of the glottis mound in the rhea [49, 58], and kiwi [55]; the shape of the caudal papillae vary with angular, rounded, and rectangular forms [55]. The lateral and dorsal projections of the arytenoid in the ostrich support the mucosal embellishments of the glottis mound, forming what has been termed a star-shape [22, 27, 50]. The lips of the glottis in both the ostrich and the rhea are supported internally by the arytenoids [22, 27, 49], however the glottis lips of the cassowary and emu [27] are not supported by the arytenoids but instead are formed of a separate mucosal structure, with a layer of dense musculature between.

### Morphological character optimisation

To assess the phylogenetic signal of syringeal, hyoidal, and laryngeal (SHL) characters, we compared their fit to morphology-only, molecular-only, and combined data trees (see methods for full details). A fair comparison of character fit across trees is difficult due to radically different taxon sampling: notably most fossil taxa are missing from the molecular-only trees. However, the most important difference in the topologies concern relationships between major clades of ratites. Hence, we used the topology of a new combined morphological and molecular data analysis as one tree for comparison; to generate the other trees, we then re-arranged the major palaeognath groups to conform to either the morphology-only tree [60], or the molecular-only tree [3] (see Methods for explanation and justification). *Lithornis* was not sampled for the molecular tree and was inserted as the most basal palaeognath based on the morphology-only analysis; however, this taxon is not codable for most SHL characters and so has very little impact on results (see Additional file 8 for results of phylogenetic analyses and character optimisation). For each topology, overall fit, as well as apomorphic and homoplasious states were identified. The results displayed in Table 1 show the syrinx, hyoid and larynx characters to have a higher congruence with the combined-data topology than either the molecular- or morphological-only topologies. The tree length is lowest (best) for the combined-data topology (101), with a higher consistency index (CI = 0.5347) and lower character homoplasy (HI = 0.4653), both leading to a higher retention index (RI = 0.6759). The number of unique and unreversed apomorphic characters (CI = 1.0) is above 10 for both the molecular-only and combined-data topologies, although the data again favours the phylogenetic relationships obtained from the combined-data (AC = 16).

**Table 1:** Optimisation of SHL palaeognath characters on morphological, molecular and combined-data topologies. Tree index statistics for each optimised topology, including CI (consistency index), HI (homoplasy index), and RI (retention index), as well as AC (unique and unreversed apomorphic characters, CI = 1.0)

Topology	Tree Length	CI	HI	RI	AC
Morphological	107	0.5047	0.4953	0.6345	8
Molecular	105	0.5143	0.4857	0.6483	14
Combined Data (Mor + Mol)	101	0.5347	0.4653	0.6759	16

**Fig. 4:**
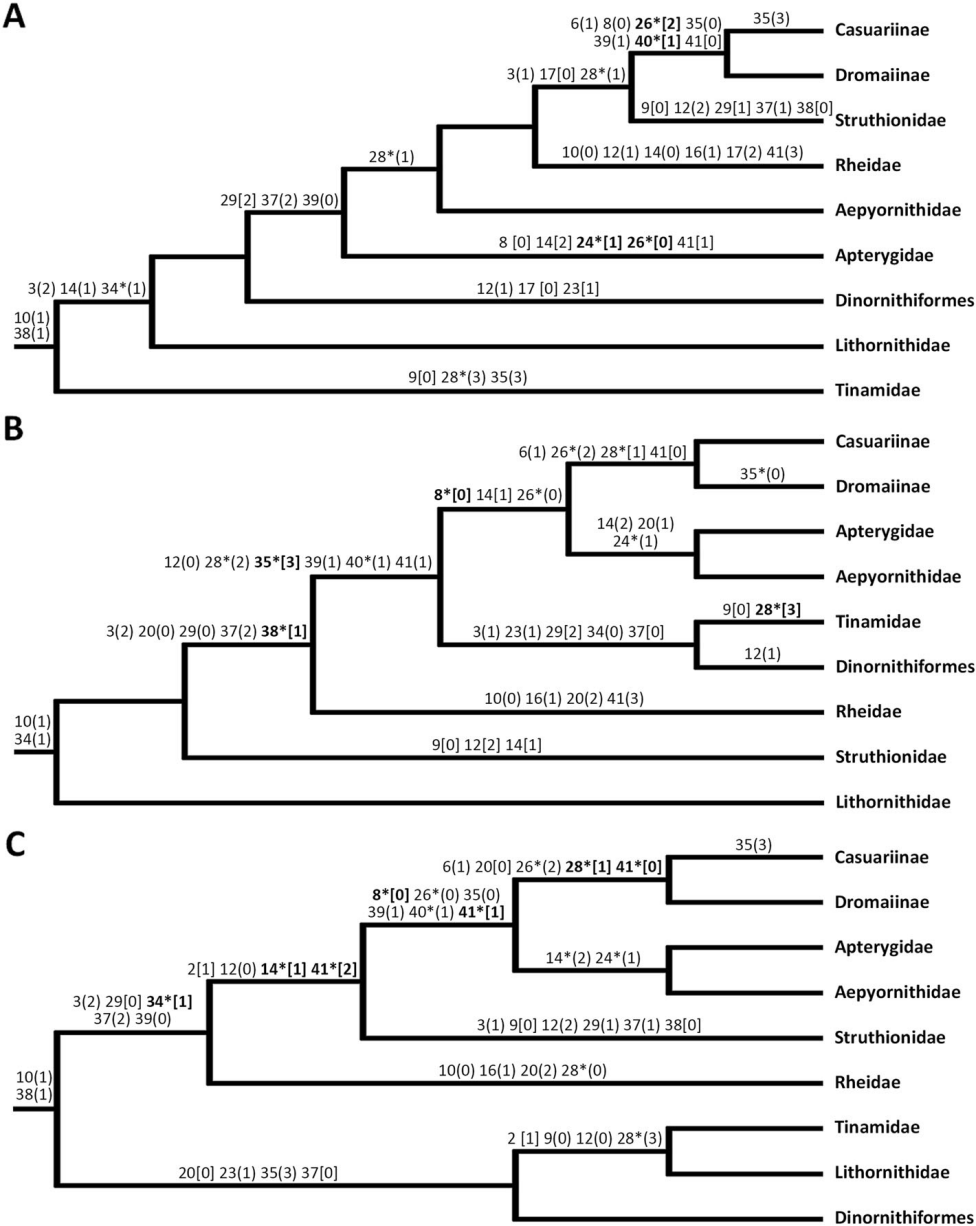
Syrinx, hyoid, and larynx character optimisation onto morphology-only, molecular-only, and combined-data topologies. Optimised characters which received a CI value of 0.5 or above are shown on each topology with the derived state in the character change denoted by brackets. Rounded brackets indicate the change was ambiguous whereas square brackets indicate unambiguous state changes. Stars show the state change to be unique and unreversed (CI=1.0) and the bold characters are both unambiguous and unique and unreversed. **a** Morphological topology optimisation. **b** Molecular topology optimisation. **c** Combined-data topology optimisation

In the following discussion and Fig. 4 above, **ambiguous** changes are those that are optimisation-dependent (e.g. vary across deltran or acctran), and **unique and unreversed characters** are those with a CI of 1. We discuss and present (Fig. 4) deltran results but flag the optimisation-dependent changes as ambiguous. The full optimisations (acctran and deltran for all three trees) are appended in the Additional file 8: SI 8.

The three topologies showed varying results for (homoplasious) autapomorphic character changes defining cassowaries. Character 11 (0 -- > 1, caudal end of the trachea almost cylindrical) was identified in both the molecular and combined-data topologies where as in both the morphological and combined-data topologies the primary autapomorphic character for the cassowary is character 35 (0 -- > 3, diamond shape, procricoid dorsal view). All identified state changes are ambiguous as a result of lack of data for *Casuarius bennetti*, (these changes might define the *C. bennetti* plus *C. casuarius* clade or *C. casuarius* alone). All three topologies share four optimised apomorphies which diagnose the cassowary/emu clade: character 5 (2 -- > 0, incomplete bronchosyringeal cartilages wider at medial ends); character 6 (0 -- > 1, syringeal asymmetry present); character 26 (morphology: 1 == > 2, molecular/combined: 0 -- > 2, numerous lingual papillae along the lateral margins of the tongue corpus); character 41 (1 == > 0, no laryngeal papillae present on the glottis lips). The character 26 state change is unique and unreversed in all topologies; the character 41 state change is only unique and unreversed in the combined-data topology.

The character states identified as apomorphic for the kiwi are optimisation ambiguous as they might apply to the kiwi/elephant bird clade or the kiwi alone; the precise branch of the state change cannot be inferred as no specimens of elephant birds preserving relevant structures are currently known. For all three topologies, only character 24 (0 -- > 1, reduced ovular, tongue shape, dorsal view) is optimised as unique and unreversed in both molecular and combined-data topologies, character 14 (1 -- > 2, no lateral intervals between cart. tracheosyringeales and cart. bronchosyringeales) was only unique and unreversed in the combined-data topology. One additional character state change was also identified in both the molecular and combined-data topologies: character 30 (morphological: 1 -- > 3, molecular/combined-data: 0 -- > 3, prominent ridges full length of cricoid corpus). The formation of the cassowary/emu and elephant bird/kiwi clade is supported by the syrinx, hyoid and larynx optimisation results. For example, character 8 (2 == > 0, tracheosyringeal rings thinner in width relative to bronchosyringeal cartilages) is both unambiguous and unique and unreversed in both the molecular and combined data topologies, there are also other supporting characters which vary between topologies (see Additional file 8: SI 8).

The tinamou-moa clade, robustly supported by DNA and combined-data but not predicted by traditional (skeletal) morphological traits, has new support from the SHL traits. Characters 23 (0 -- > 1, epibranchials shorter than ceratobranchials), and 37 (molecular: 2 == > 0, combined-data: 1 == > 0, ossification of the arytenoids) optimised for both the molecular and combined topologies with further characters varying between the two topologies based on the placement of the clade within the palaeognath lineage. Character 29 (0 == > 2, ossified cricoid cartilage) was identified as apomorphic for moa/tinamou only in the molecular topology. This is a result of the basal divergence of the moa/tinamou clade in the combined-data topology, leading to an alternative state for this character (2 == > 0, cartilaginous cricoid) optimising as an apomorphy for the clade containing all other crown palaeognaths. The occurrence of ossification of the cricoid and arytenoids in outgroup taxa such as *Grus, Gallus* and *Anseranas*, as well as in moa and tinamou, suggest state 2 is ancestral, shared with both palaeognaths and neognaths. This identified link between the tinamou/moa clade and many outgroup taxa shows potential support for the basal tinamou/moa divergence within the combined-data topology with characters 37 and 29 retained from the last common ancestor between palaeognaths and neognaths; alternatively, it might be an indication of increased structural support requirements [61], rather than true descent. However, two recent studies using phylogenomic methodologies on broad scale avian and palaeognath topologies, placed the tinamou/moa clade low in the palaeognath lineage [62, 63] as the sister group to palaeognaths other than *Struthio*. Both studies prioritised molecular, especially genomic data and thus, morphological convergence should have had limited influence. The results of these two studies, as well as the SHL optimisation results, support an earlier moa and tinamou divergence than in the molecular topology used here and suggest the rhea is sister to the elephant bird/kiwi plus cassowary/emu clade; Cloutier et al. [63] found no support for the rhea to be sister to all non-ostrich palaeognaths, as suggested by Grealy et al. [3].

In addition, a cranial character, character 43 (state 1, articulation of the maxillary process of the nasal with the maxilla) was identified as an apomorphic character for the tinamou and moa through a review of the literature [64] and included in the morphological data matrix, although this was not included in the optimisation tests as these were specifically meant to evaluate the support of SHL characters for alternative phylogenies.

In the molecular topology, character 31 changes for palaeognaths (0 -- > 3, proc. rostralis absent) then changes again after the rhea and ostrich diverge (3 -- > 2, proc. present but reduced), suggesting support for a basal divergence of the rhea and ostrich. Six other characters also support the basal divergence of the rhea and ostrich, with state changes above the rhea and ostrich divergences: i.e. characters 35 (1 == > 3, diamond procricoid shape) and 40 (0 -- > 1, arytenoid cartilage a separate structure to the glottis lips) both unique and unreversed. However, primarily for character 31, the state changes have low CI values and there is no suggestion within the optimisation results for the morphological and combined-data topologies that a later divergence of the rhea and ostrich, as in the combined topology, is less supported by the SHL characters.

## Discussion

### Functional morphology of the syrinx, hyoid, and larynx in palaeognaths

#### Syrinx

In comparison to most neognaths and other palaeognaths, the syrinx of the cassowary is poorly developed and simple in structure. The implications of this are poorly understood however, as the location of the syrinx deep within the body has restricted functional research [65], and the state of the ancestral syrinx remains unknown [35]. In spite of this, the simplicity of the cassowary syrinx in comparison to non-palaeognath taxa and the rhea, which produce a broader range of vocalisations, supports a direct relationship between anatomical complexity and vocal virtuosity [66].

The presence of structures such as the lateral tympaniform membrane and intrinsic musculature have previously been correlated with a broad vocal repertoire. For example, the presence of the lateral tympaniform membrane in the tinamou has been linked to the whistle-like notes, flute-like trill and alert or disturbed peeping calls they make [40]. Intrinsic musculature, arising and inserting within the syrinx, directly acts upon the syrinx to increase control over elements and allow for a greater vocabulary [66, 67]. Rhea chicks develop broad repertoires, consisting of around five vocalisation types, although this diminishes with age. Vocalisation acts as the primary form of communication in chicks as it is better suited to the ecological contingencies experienced during juvenile life stages, and thus, intrinsic muscles and increased syringeal complexity reflect this requirement [68]. Kiwi with well-developed syringeal intrinsic musculature communicate with sexually dimorphic calls over large distances at night and produce a variety of distinctive calls [69].

A pessulus supports the syringeal membranes at the point of tracheal bifurcation, reducing chances of dorso-ventral collapse [17]. Although the pessulus is characteristic of the tracheo-bronchial syrinx and exists both in passerine and non-passerine taxa, its absence is not rare ([19], p., 141, [34, 60]). Originally the pessulus and associated semilunar membrane were assumed to play some role in sound production, although its experimental removal resulted in no noticeable modification to sound production, suggesting structural support to be the only functional benefit [70, 71]. Its absence in the cassowary and emu is likely correlated with a sparse vocal repertoire [18], and its presence in rhea [18] with complex vocalisations- at least in young. This does not explain however, the absence of the pessulus in the tinamou [40] and kiwi [18, 21], both of which have more complex vocalisations than rheas.

Ossification of the syrinx is common among neognaths, enhancing strength and resistance of syringeal structures during vocalisation, when the potential muscular force applied is at its peak intensity [34]. Ossification may also allow for increased frequency – production of more rapid song elements – with efficient modulation and precise temporal control [34]. Such rapid movement and strength of syringeal elements is not required for the basic vocalisations produced by cassowaries, and therefore ossification of syringeal elements is absent. Ossification of syringeal elements occurs in moa and, alongside the presence of the pessulus, may indicate a requirement for strengthening of the syrinx, thus suggesting a broader vocal repertoire.

#### Hyoid

Morphological similarity of hyoid elements between the cassowary and emu was expected due to their well-established close relationship. Despite this, the hyoid apparatus in the cassowary and emu differ, primarily in relative length of structures; i.e. the cassowary ceratobranchials are elongate compared to the epibranchials, while in the emu both structures have similar length. This is possibly a reflection of the different feeding strategies between the two taxa, with studies finding evidence for a strong functional relationship between feeding mode and cranio-lingual morphology [72, 73]. Other than the frugivorous cassowary, all other palaeognaths have a varied diet, comprised of leaves, flowers, fruits and grass seeds, as well as dicot herbs and shrubs [74]. Often various insects and small animals are also included in the diets of most palaeognaths, particularly the kiwi, primarily feeding on soil invertebrates [75–77].

Elongation of hyoid elements in Aves is directly associated with tongue protrusion and its increased control, with extreme morphologies noted for the taxa such as woodpeckers and hummingbirds ([23], p., 53, [78]). The cassowary employs the ‘catch and throw’ feeding method; a method of feeding which requires a reduced tongue to prevent injury during the feeding process [28, 79]. For this method there is no requirement for tongue protrusion, and therefore, it is likely that the minor elongation of the cassowary ceratobranchials may be associated with control of the tongue, allowing the cassowary to arrange the tongue in a position that doesn’t inhibit consumption.

Ossification of avian hyoid elements is relatively consistent among taxa ([23], p., 52, [24], p., 362, [80]) and has been linked to the attachment of muscles associated with coordination of hyoid movement primarily during feeding [48]. Nonetheless, no association between basiurohyal ossification and diet within palaeognaths has been identified with limitations concerning variation between closely related taxa where the diet is similar [72]. The cassowary and ostrich basiurohyals are cartilaginous, whereas ossification is present in tinamou and moa. The tinamou and ostrich are omnivores, the moa was an herbivore and the cassowary is a specialist frugivore [53]. Therefore, it is likely that hyoid element ossification in palaeognaths is not directly related to function and may instead be correlated with evolutionary relationships.

#### Larynx

The avian larynx has previously received little attention, with the functional purpose and significance of the morphology and ossification of elements only briefly reported. Analyses which assess the structure and functional significance of the larynx in Aves primarily focus on the associated musculature which, although functionally important, provide little insight into the role of the skeletal morphology. The ontogenetic rate of ossification in the larynx differs by individual element and by species, with findings suggesting a heavy influence from the size of the species and age of the individual ([25, 26], p., 74). However, without analysis of numerous individuals or complex ontogenetic studies, the effect of age on ossification cannot be determined. Aside from age, ossification of laryngeal elements such as the cricoid of the moa and tinamou has been explained by Hogg [61] as a supplementary modification to increase support against collapse. Hogg [61] did concede however that determining the necessity of ossification in the trachea and larynx was problematic, and no comparison was made between his study taxa, domestic fowl, and taxa with similar ecologies which lack an ossified larynx. Various differences in the feeding ecologies and body size of the moa and tinamou do not assist in determining the functional significance of ossification of elements within clade. In comparison to mammalian counterparts, the avian trachea is well adapted to reducing chances of collapse with the presence of complete rings, and ossification is potentially a supplementary adaptation to assist with this [61]. If ossification is not an ancestral avian character linking tinamou and moa and other outgroup taxa, but a derived character, further investigation into the morphology of moa and tinamou, and other neognath taxa which developed ossified cricoids, may find drivers in behaviour related to the ossified state.

### Morphological support for Palaeognath molecular phylogeny

The results of the optimisation of syringeal, hyoidal and laryngeal characters onto the morphological, molecular and combined-data topologies suggests these structures might provide novel phylogenetic information. The results outlined in Table 1 show the morphological data for the syrinx, hyoid and larynx structures in palaeognaths had the highest concordance with the combined-data topology, followed closely by the molecular topology. Interestingly, the morphological topology was least preferred. This indicates the convergent morphology for flightlessness and large body size within palaeognaths, linked to confounding morphology-based topologies, is not greatly influencing their syringeal, hyoidal, or laryngeal morphology, and thus these characters potentially provide more reliable phylogenetic information.

As no syrinx, hyoid or larynx elements have been recovered for the any aepyornithids, the results of the study shed no new morphological light on the molecular kiwi/elephant-bird clade. However, apomorphic character states currently known only in the kiwi may be apomorphic for the kiwi/elephant-bird clade. To test this, such structures would need to be recovered for the elephant bird. This is unlikely however: the cartilaginous state of these structures in the kiwi suggest they are also cartilaginous in elephant birds, which is consistent with their absence in the fossil record. If these elements were consistently ossified, they would likely have been identified, as in the moa.

The new SHL characters shared by the tinamou and moa provides novel morphological support for the sister placement of these two taxa in molecular and combined-data topologies. This is important as morphological apomorphic characters supporting this clade have been elusive. Additionally, a cranial character not included in previous phylogenetic analyses, is newly recognised as a synapomorphy for the moa/tinamou clade: presence of the articulation of the maxillary process of the nasal with the maxilla (see [64]).

## Conclusion

The morphology of the syrinx, hyoid and larynx of the cassowary show close similarity to that of the emu, supporting their well-resolved placement as sister taxa. Substantial variation across palaeognaths for these structures was identified and described, forming the basis for new phylogenetic analyses in combination with existing morphological and molecular data. Novel morphological synapomorphies were also identified for the moa and tinamou clade; morphological support for the molecular evidence uniting these two taxa has previously remained elusive. The phylogenetic signal of syringeal, hyoidal, and laryngeal morphology suggests these structures are more informative of relationships among palaeognath taxa compared to post-cranial characters. This is important as all ratite palaeognaths have convergence in their morphology due to flightlessness and large body size that has hitherto obscured evolutionary relationships. Many syringeal, hyoidal, and laryngeal characters were identified which supported topological relationships found in the molecular and combined-data analyses that are absent in the morphology-only phylogeny, such as the tinamou/moa clade, and the kiwi/elephant bird, cassowary/emu clade.

## Methods

### Specimen

The cassowary analysed in this study was an adult female acquired from the Department of Environment and Heritage Protection, Innisfail, Queensland. Death occurred on June 9th, 2016, when the cassowary was hit by a vehicle, south of Tully, Queensland. The complete ossification and synostosis of all compound elements including the skull, presence of fully developed ovaries, and a weight of over 40 kg led to the conclusion that the cassowary was a fully mature adult female. The frozen cassowary was transported to Flinders University on permit number I13689, issued 16th January 2017. Its skeleton is catalogued as FUR180 in the Flinders University Palaeontology Collection.

### Scanning and Modelling

Following the methodology of Clement et al. [81], the hyoid apparatus, larynx and syrinx were extracted as a single element from the cassowary body, and dehydrated in increasingly strong concentrations of ethanol (C_2_H_6_O), 70 and 85%, prior to being placed in an iodine, ethanol solution formed from 2 L of 100% ethanol and 200 g of iodine (Iodine ACS reagent > 99.8 solid). The contrast agent iodine was selected for its differential affinities to the major soft tissue types, and safety of use [82–84]. Contrast agents differentially stain soft tissue types making visible increased contrast between tissues enabling higher levels of detail of morphology, organisation, and arrangement to be captured when CT or μCT scanned [84–86].

The specimen was μCT scanned on the 11th May 2018 at Adelaide Microscopy (the University of Adelaide). A 2006 Skyscan-1076 in vivo x-ray microtomograph (Bruker Micro CT, Kontich, Belgium) machine was used to scan the specimen, with a resultant pixel size of 35 μm. The resulting image collections from the full scans were amalgamated into volume images. 3D modelling and segmentation of the μCT volume images was conducted through thresholding and segmentation in Mimics [87] to produce clean, reliable 3D models of skeletal elements. Elements were modelled and edited individually, prior to being reconstructed as a single colume. For a 3-dimensional visual of the three modelled elements of ths outhern cassowary, see Additional file 5: SI 5.

### Description and comparative analysis

Comparative anatomy examination of the cassowary specimen was completed based on interpretation of elements from the literature, primarily syrinx and larynx descriptions from King [19] and McLelland [26] respectively, with nomenclature derived from Baumel et al. [88]. The morphologies of comparative species were also derived from the literature, athough information was lacking for some palaeognaths, primarily kiwi, moa and tinamou, and a complete absence is noted for elephant birds. Data on the cricoid of the kiwi was obtained from Catherine Tate, Dr. Jean-Claude Stahl, and Alan Tennyson of the Museum of New Zealand Te Papa Tongarewa. Laryngeal and hyoid element data for three tinamou species was also obtained from Marcus Cenizo of the Museo de Historia Natural de La Pampa. All available moa specimens in the Museum of New Zealand Te Papa Tongarewa, wherein presence of tracheal ring sets predicated the possible presence of syringeal and laryngeal structures, were examined by PM.

### Phylogenetics

#### SHL morphological characters, general morphological characters, and molecular data

Forty-two SHL characters (Additional file 2: SI 2) and an additional cranial character (see below) were coded in Mesquite [89] for 21 palaeognath species representing eight families and six outgroup taxa (Additional file 4: SI 4). Characters were developed from the analysis of syrinx, hyoid, and larynx morphology among Palaeognathae and outgroup taxa, as well as from characters used in previous studies [i.e.^18^, ^35^, ^90^]. The six outgroup species were coded from the available literature, as well as reference specimens. This includes the crane, *Grus rubicunda*; coded for all non-SHL morphological characters by Trevor Worthy. Palaeognath and outgroup species from Worthy and Scofield [60] were selected to allow comparison with previous analyses and for incorporation into future morphological analyses. An additional character concerning the articulation of the maxillary process of the nasal bar with the maxilla was developed from Mayr [64]. The 42 SHL characters and a new maxillary character were added to characters 1–179 from Worthy and Scofield [60], to generate a total data set of 222 characters. Morphological characters were ordered when appropriate (e.g. morphoclines), with ordering of skeletal characters as in Mitchell et al. [39]. In the combined dataset, molecular data from Grealy et al. [3] was included (file mt_nuc_nt.phy from RaxML_inputs.zip) and corresponds to characters 1–27,116. Characters 27,117–27,295 correspond to characters 1–179 from Worthy and Scofield [60] and characters 27,296–27,338 to SHL characters 180–222. Characters 1–27,116 in the combined data matrix correspond to 1–27,116 in Grealy et al. [3].

### Phylogenetic analyses

We used both morphological and molecular phylogenies to assess the phylogenetic utility of the syringeal, hyoidal, and laryngeal structures discussed above. We analysed our above data in two ways, to produce morphological and combined-data topologies on which the syrinx, hyoid and larynx characters could be optimised. We also used an existing molecular phylogeny for the same optimisations.

Bayesian inference was selected for the combined-data analysis as model-based methods are generally considered more reliable than parsimony for molecular data, which form the bulk of the combined-data matrix. The topology was inferred through the Markov Chain Monte Carlo (MCMC) procedures in the program MrBayes ([91], version 3.2.2), and implemented through the CIPRES Scientific Gateway ([92], version 3.3) (Additional file 6: SI 6.1). Bayesian analysis was run with a burn in fraction of 25% for 15 million generations, sampling every 10000 generations, and employing four runs each with four chains (one cold and three incrementally heated). The analysis employed the PartitionFinder [93] best fit substitution model for each subset, with the temperature of the MCMC analysis set to 0.08. Molecular subset branch lengths were linked and scaled by substitution rate.

The morphological topology was produced through parsimony phylogenetic analyses conducted in PAUP* [94] and implemented through CIPRES Scientific Gateway ([92], version 3.3) (Additional file 6: SI 6.2). Taxa with no morphological data were excluded from the analysis, which was run with the heuristic algorithm and 5000 random addition replicates per search, using tree-bisection-reconnection (TBR) branch-swapping. The random stepwise-addition option was selected. Bootstrap analysis [95] was also utilised to estimate nodal support using the heuristic search methods with 500 replicates. Trees were rooted by the 6 outgroup taxa; all characters were weighted equally.

The molecular topology also used within this study was extracted from Grealy et al. [3], the source for the molecular data which contributed to the combined-data topology as stated above. For optimisation of characters, discussed below, this topology was modified to include species without molecular data. Their placement was based on their inferred relationships from morphological data. Only outgroup taxa with morphological data from the two previously discussed analyses were included in the optimisation as we required a consistent outgroup and are only addressing the fit of characters for palaeognath taxa.

#### Character optimisation

The three above phylogenies provide hypotheses which can be tested against the new syrinx, hyoid and larynx (SHL) data. Optimisation of the 42 SHL characters (the maxillary character, character 43, was excluded) was performed using parsimony methods in PAUP* [94], under both accelerated transformation (ACCTRAN) and delayed transformation (DELTRAN); the fit of the SHL characters against each tree was addressed using total tree length, as well as ensemble consistency and retention indices. The SHL characters were optimised onto the bootstrap tree for the morphological topology. Eighteen characters were ordered as noted in Additional file 3: SI 3. Unambiguous and unique and unreversed characters were identified as important for their contribution to defining individual clades (Additional file 7: SI 7). For complete results please refer to Additional file 8: SI 8.

## Supplementary information

**Additional file 1: SI 1.** Moa species by taxon with tracheal rings. All listed specimens had tracheal rings preserved; specimens with bronchosyringeal half-rings and syringeal keeled rings are specifically identified. A list of all moa taxa with recovered tracheal rings in the Museum of New Zealand Te Papa Tongarewa.

**Additional file 2: SI 2.** Tinamou cricoid images from Museo de Historia Natural de La Pampa. Images of the larynx and cricoid of three tinamou species (*Nothura maculosa, Eudromia elegans,* and *Rhynchotus rufescens*), taken by Marcos Cenizo, Museo de Historia Natural de La Pampa.

**Additional file 3: SI 3.** Syrinx, hyoid, and larynx characters and character states. All characters developed through morphological analysis of the syrinx, hyoid and larynx in palaeognaths, used in the phylogenetic analyses and optimised onto the three resulting topologies.

**Additional file 4: SI 4.** Character matrix for 28 palaeognath and outgroup taxa, 42 SHL characters and one maxillary character. Character matrix for all 28 palaeognath and outgroup taxa and 42 characters assessed in this study.

**Additional file 5: SI 5.** Video footage of 3-dimensional models for the syrinx, hyoid, and larynx of the Southern Cassowary. Three individual short videos of the 3D models of the syrinx, hyoid, and larynx. The images turn on a single axis to show the structures from various angles.

**Additional file 6: SI 6.** Input data for two phylogenetic analyses. Two text files. The first file (SI 6.1) includes the complete input file with all included data for the combined phylogenetic analysis conducted using Bayesian methods. The second file (SI 6.2) includes the input for the parsimony phylogenetic analysis of morphological data.

**Additional file 7: SI 7.** Input data for the optimisation character analyses. Complete input file for the optimisation analyses which were run in the program PAUP* [89], with coding for all three tested topologies.

**Additional file 8: SI 8.** Optimisation Results. Results for optimisation analyses of SHL data onto three topologies.

## Data Availability

All data generated or analysed during this study are included in this published article and its supplementary information files.

## Abbreviations

FU: Flinders University, Bedford Park (5042), South Australia, Australia
MMC: Marcos Cenizo Personal Collection, Santa Rosa (6300), La Pampa, Argentina
NMNZ: Museum of New Zealand Te Papa Tongarewa, Wellington (6011), New Zealand

## References

[1] Hackett SJ, Kimball RT, Reddy S, Bowie RCK, Braun EL, Braun MJ (2008). A phylogenomic study of birds reveals their evolutionary history. Science.

[2] Harshman J, Braun EL, Braun MJ, Huddleston CJ, Bowie RCK, Chojnowski JL (2008). Phylogenomic evidence for multiple losses of flight in ratite birds. PNAS.

[3] Grealy A, Phillips M, Miller G, Gilbert MTP, Rouillard JM, Lambert D (2017). Eggshell palaeogenomics: Palaeognath evolutionary history revealed through ancient nuclear and mitochondrial DNA from Madagascan elephant bird (*Aepyornis* sp.) eggshell. Mol Phylogenet Evol.

[4] Sibley CG, Ahlquist JE (1990). Phylogeny and classification of birds.

[5] Phillips MJ, Gibb GC, Crimp EA, Penny D (2010). Tinamous and moa flock together: mitochondrial genome sequence analysis reveals independent losses of flight among ratites. Syst Biol.

[6] Johnston P (2011). New morphological evidence supports congruent phylogenies and Gondwana vicariance for palaeognathous birds. Zool J Linnean Soc.

[7] Yonezawa T, Segawa T, Mori H, Campos PF, Hongoh Y, Endo H (2017). Phylogenomics and morphology of extinct palaeognaths reveal the origin and evolution of the ratites. Curr Biol.

[8] Dickinson EC, Remsen JV (2013). The Howard & Moore complete checklist of the birds of the world.

[9] Davies SJJF, Davies SJJF (2002). Cassowaries. Ratites and Tinamous: Tinamidae, Rheidae, Dromaiidae, Casuariidae, Apterygidae, Struthionidae.

[10] Bradford MG, Westcott DA (2010). Consequences of southern cassowary (*Casuarius,* L.) gut passage and deposition pattern on the germination of rainforest seeds. Austral Ecol.

[11] Abourachid A, Renous S (2000). Bipedal locomotion in ratites (Palaeognathiform): examples of cursorial birds. IBIS.

[12] Naish D, Perron R (2016). Structure and function of the cassowary’s casque and its implications for cassowary history, biology, and evolution. Hist Biol.

[13] Ryan M, Brenowitz E (1985). The role of body size, phylogeny, and ambient noise in the evolution of bird song. Am Nat.

[14] Podos J, Huber SK, Taft B (2004). Bird song: the interface of evolution and mechanism. Annu Rev Ecol Evol Syst.

[15] Podos J, Nowicki S (2004). Beaks, adaptation, and vocal evolution in Darwin’s finches. BioScience.

[16] Mason NA, Burns KJ, Tobias JA, Claramunt S, Seddon N, Derryberry EP (2016). Song evolution, speciation, and vocal learning in passerine birds. Evolution.

[17] Cevik-Demirkan A, Haziroglu RM, Kurtul I (2007). Gross morphological and histological features of larynx, trachea and syrinx in Japanese quail. Anat Histol Embryol.

[18] Forbes W. A. (2009). On the Conformation of the Thoracic End of the Trachea in the“Ratite”Birds. Proceedings of the Zoological Society of London.

[19] King AS, King AS, McLelland J (1989). Functional anatomy of the syrinx. Form and function in birds.

[20] Beddard F (1898). The structure and classification of birds.

[21] Pycraft W. On the morphology and phylogeny of the Palaeognathae (Ratitae and Crypturi) and Neognathae (Carinatae). Trans Zool Soc Lond. 1900;15:149–290.

[22] Soley JT, Tivane C, Crole MR (2015). Gross morphology and topographical relationships of the hyobranchial apparatus and laryngeal cartilages in the ostrich (*Struthio camelus*). Acta Zool.

[23] Homberger DG, Maina J (2017). The avian lingual and laryngeal apparatus within the context of the head and jaw apparatus, with comparisons to the mammalian condition: functional morphology and biomechanics of evaporative cooling, feeding, drinking, and vocalisation. The biology of the avian respiratory system: evolution, development, structure and function.

[24] Tomlinson CAB. Feeding in Palaeognathous birds. In: Schwenk K, editor. Feeding: form and function in tetrapod vertebrates. San Diego: Academic Press; 2000. p. 359–94.

[25] Zweers GA, van Pelt HC, Beckers A (1981). Morphology and mechanics of the larynx of the pigeon (*Columba livia* L.): a drill-chuck system (Aves). Zoomorphology.

[26] McLelland J, King AS, McLelland J (1989). Larynx and trachea. Form and function in birds.

[27] Crole MR, Soley JT (2012). What prevents *Struthio camelus* and *Dromaius novaehollandiae* (Palaeognathae) from choking? A novel anatomical mechanism in ratites, the linguo-laryngeal apparatus. Front Zool.

[28] Erdogan S, Iwasaki S (2014). Function-related morphological characteristics and specialised structures of the avian tongue. Ann Anat.

[29] Crole MR, Soley JT (2009). Morphology of the tongue of the emu (*Dromaius novaehollandiae*). I. Gross anatomical features and topography. Onderstepoort J Vet Res.

[30] Mauricio GN, Areta JI, Bornschein MR, Reis RE (2012). Morphology-based phylogenetic analysis and classification of the family Rhinocryptidae (Aves: Passeriformes). Zool J Linnean Soc.

[31] Hughes JM (2000). Monophyly and phylogeny of cuckoos (Aves, Cuculidae) inferred from osteological characters. Zool J Linnean Soc.

[32] Livezey BC, Zusi RL (2007). Higher-order phylogeny of modern birds (Theropoda, Aves: Neornithes) based on comparative anatomy. II. Analysis and discussion. Zool J Linnean Soc.

[33] Manegold A (2008). Morphological characters of the tongue skeleton reveal phylogenetic relationships within the Corvidae (Oscines, Passeriformes). Emu-Austral Ornithology.

[34] During DN, Ziegler A, Thompson CK, Ziegler A, Faber C, Muller J (2013). The songbird syrinx morpheme: a three-dimensional, high-resolution, interactive morphological map of the zebra finch vocal organ. BMC J Biol.

[35] Clarke JA, Chatterjee S, Li Z, Riede T, Agnolin F, Goller F (2016). Fossil evidence of the avian vocal organ from the Mesozoic. Nature.

[36] Parker WK (1866). VII. On the structure and development of the skull in the ostrich tribe. Philos Trans R Soc.

[37] Gadow H, Selenka E (1891). Bronn's Klassen und Ordnungen des Thier-Reichs. Anatomischer Theil Leipzig.

[38] Livezey BC (1998). A phylogenetic analysis of the Gruiformes (Aves) based on morphological characters, with an emphasis on the rails (Rallidae). Philos Trans R Soc Lond B.

[39] Mitchell KJ, Llamas B, Soubrier J, Rawlence NJ, Worthy TH, Wood J (2014). Ancient DNA reveals elephant bird and kiwi are sister taxa and clarifies ratite bird evolution. Science.

[40] Garitano-Zavala A (2009). Evolutionary loss of the extrinsic syringeal muscle sternotrachealis in Darwin's Nothura (*Nothura darwinii*) - Pérdida evolutiva del músculo siríngeo extrínseco esternotraqueal en *Nothura darwinii*. Auk.

[41] Fowler ME (1991). Comparative clinical anatomy of ratites. J Wildl Med.

[42] Yildiz H, Bahadir A, Akkoc A (2003). A study on the morphological structure of syrinx in ostriches (*Struthio camelus*). Anat Histol Embryol.

[43] Picasso MBJ, Carril J (2013). The peculiar syrinx of *Rhea americana* (greater rhea, Palaeognathae). Vertebr Zool.

[44] Oliver WRB. The moas of New Zealand and Australia. Wellington: Dominion Museum Bulletin; 1949.

[45] Owen R. Brain, larynx, and trachea in the genus *Dinornis*. Trans Zool Soc. 1871;7:385–92.

[46] Worthy TH, Holdaway RN. The lost world of the moa: Prehistoric life of New Zealand. Indiana: Indiana University Press; 2002.

[47] Tadjalli M, Mansouri SH, Poostpasand A (2008). Gross anatomy of the oropharyngeal cavity in the ostrich (*Struthio camelus*). Iran J Vet Res.

[48] Li Z, Zhou Z, Clarke JA (2018). Convergent evolution of a mobile bony tongue in flighted dinosaurs and pterosaurs. PLoS One.

[49] Crole MR, Soley JT (2012). Gross anatomical features of the tongue, lingual skeleton and laryngeal mound of *Rhea americana* (Palaeognathae, Aves): morpho-functional considerations. Zoomorphology.

[50] Faraggiana R (1933). Sulla morfologia della lingua e del rialzo laringeo di alcune specie di Ucc. Boll Musei Zool Anat Comp Della.

[51] Jackowiak H, Ludwig M (2008). Light and scanning electron microscopic study of the structure of the ostrich (*Struthio camelus*) tongue. Zool Sci.

[52] Maxwell EE (2009). Comparative ossification and development of the skull in palaeognathous birds (Aves: Palaeognathae). Zool J Linnean Soc.

[53] Bradford MG, Westcott DA (2011). Predation of cassowary dispersed seeds: is the cassowary an effective disperser?. Integr Zool.

[54] Crole MR, Soley JT (2010). Gross morphology of the intra-oral rhamphotheca, oropharynx and proximal oesophagus of the emu (*Dromaius novaehollandiae*). Anat Histol Embryol.

[55] McCann C (1973). The tongues of kiwis (*Apteryx* spp.). Notornis.

[56] Sales J (2006). Feeding of the captive kiwi. Zoos’ Print J.

[57] Cunningham SJ, Corfield JR, Iwaniuk AN, Castro I, Alley MR, Birkhead TR (2013). The anatomy of the bill tip of kiwi and associated somatosensory regions of the brain: comparisons with shorebirds. PLoS One.

[58] Rodrigues MN, Tivane CN, Carvalho RC, Olivera GB, Silva RSB, Ambrosio CE (2012). Gross morphology of the rhea oropharyngeal cavity. Pesqui Vet Bras.

[59] Scarlett R (1975). Thyroid bones of moas. Rec Canterbury Mus.

[60] Worthy TH, Scofield RP (2012). Twenty-first century advances in knowledge of the biology of the moa (Aves: Dinornithiformes): a new morphological analysis and moa diagnosis revised. N Z J Zool.

[61] Hogg DA (1982). Ossification of the laryngeal, trachea and syringeal cartilages in the domestic fowl. J Anat.

[62] Kimball Rebecca T., Oliveros Carl H., Wang Ning, White Noor D., Barker F. Keith, Field Daniel J., Ksepka Daniel T., Chesser R. Terry, Moyle Robert G., Braun Michael J., Brumfield Robb T., Faircloth Brant C., Smith Brian Tilston, Braun Edward L. (2019). A Phylogenomic Supertree of Birds. Diversity.

[63] Cloutier Alison, Sackton Timothy B, Grayson Phil, Clamp Michele, Baker Allan J, Edwards Scott V (2019). Whole-Genome Analyses Resolve the Phylogeny of Flightless Birds (Palaeognathae) in the Presence of an Empirical Anomaly Zone. Systematic Biology.

[64] Mayr Gerald (2018). Comparative morphology of the avian maxillary bone (os maxillare) based on an examination of macerated juvenile skeletons. Acta Zoologica.

[65] Elemans CP, Muller M, Larson ON, van Leeuwen JL (2009). Amplitude and frequency modulation control of sound production in a mechanical model of the avian syrinx. J Exp Biol.

[66] Gaunt AS (1983). An hypothesis concerning the relationship of syringeal structure to vocal abilities. Auk.

[67] Gaunt AS, Gaunt SL (1985). Electromyographic studies of the syrinx in parrots (Aves, Psittacidae). Zoomorphology.

[68] Beaver PW (1978). Ontogeny of vocalisation in the greater Rhea. Auk.

[69] CORFIELD JEREMY, GILLMAN LEN, PARSONS STUART (2008). VOCALIZATIONS OF THE NORTH ISLAND BROWN KIWI (APTERYX MANTELLI). The Auk.

[70] Miskimen M (1951). Sound production in passerine birds. Auk.

[71] Gaunt AS, Wells MK (1973). Models of syringeal mechanisms. Am Zool.

[72] Gardner LL (1925). The adaptive modifications and the taxonomic value of the tongue in birds. Proc United States Natl Mus.

[73] Li Z, Clarke JA (2016). The craniolingual morphology of waterfowl (Aves, Anseriformes) and its relationship with feeding mode revealed through contrast-enhanced X-ray computed tomography and 2D morphometrics. Evol Biol.

[74] Wood JR, Rawlence NJ, Rogers GM, Austin JJ, Worthy TH, Cooper A (2008). Coprolite deposits reveal the diet and ecology of the extinct New Zealand megaherbivore moa (Aves, Dinornithiformes). Quat Sci Rev.

[75] Marchant S, Higgins PJ. Handbook of Australian, New Zealand & Antarctic Birds. Volume 1. Part a: ratites to petrels. Melbourne: Oxford University Press; 1990.

[76] Noble JC (1991). On ratites and their interactions with plants. Rev Chil Hist Nat.

[77] Mosa SG (1993). Fall and winter diet and habitat preferences of the Andean tinamou (*Nothura pentlandii*) in the Northwest Argentina. Stud Neotropical Fauna Environ.

[78] Jung JY, Naleway SE, Yaraghi N, Herrera S, Sherman V, Bushong EA (2016). Structural analysis of the tongue and hyoid apparatus in a woodpecker. Acta Biomater.

[79] Erdogan S, Alan A (2012). Gross anatomical and scanning electron microscopic studies of the oropharyngeal cavity in the European magpie (*Pica pica*) and the common raven (*Corvus corax*). Microsc Res Tech.

[80] Korzun LP, Érard C, Gasc J (2004). Morphofunctional study of the bill and hyoid apparatus of *Momotus momota* (Aves, Coraciiformes, Momotidae): implications for omnivorous feeding adaptation in motmots. Biol Pathol Anim.

[81] Clement AM, Nysjö J, Strand R, Ahlberg PE (2015). Brain – endocast relationship in the Australian lungfish, *Neoceratodus forsteri*, elucidated from tomographic data (Sarcopterygii: Dipnoi). PLoS One.

[82] Metscher BD (2009). MicroCT for comparative morphology: simple staining methods allow high-contrast 3D imaging of diverse non-mineralised animal tissues. BMC Physiol.

[83] Degenhardt K, Wright AC, Horng D, Padmanabhan A, Epstein JA (2010). Rapid 3D phenotyping of cardiovascular development in mouse embryos by micro-CT with iodine staining. Circ Cardiovasc Staining.

[84] Gignac PM, Kley NJ, Clarke JA, Colbert MW, Morhardt AC, Cerio D (2016). Diffusible iodine-based contrast-enhanced computed tomography (diceCT): an emerging tool for rapid, high-resolution, 3-D imaging of metazoan soft tissues. J Anat.

[85] Gignac PM, Kley NJ (2014). Iodine-enhanced micro-CT imaging: methodological refinements for the study of the soft-tissue anatomy of post-embryonic vertebrates. J Exp Zool.

[86] Thorn C, Sedlmayr J, McNulty M. Utilising iodine to image alligator soft tissue structures with Micro-CT. FASEB J. 2015;29(suppl. 1):868.

[87] Materialise (2017). Mimics version 18.0, 3D medical image processing software.

[88] Baumel JJ, King AS, Breazile JE, Evans HE, Vanden Berge JC, editors. Handbook of avian anatomy: Nomina Anatomica Avium. 2^nd^ ed. Massachusetts: Publications of the Nuttall Ornithological Club; 1993.

[89] Maddison WP, Maddison DR (2018). Mesquite: a modular system for evolutionary analysis. Version 3.40.

[90] Griffiths Carole S. (1994). Syringeal Morphology and the Phylogeny of the Falconidae. The Condor.

[91] Ronquist F., Huelsenbeck J. P. (2003). MrBayes 3: Bayesian phylogenetic inference under mixed models. Bioinformatics.

[92] Miller MA, Pfeiffer W, Schwartz T. Creating the CIPRES Science Gateway for inference of large phylogenetic trees. New Orleans: Proceedings of the Gateway Computing Environments Workshop (GCE); 2010. p. 1–8.

[93] Lanfear R., Calcott B., Ho S. Y. W., Guindon S. (2012). PartitionFinder: Combined Selection of Partitioning Schemes and Substitution Models for Phylogenetic Analyses. Molecular Biology and Evolution.

[94] Swofford DL. PAUP*. Phylogenetic analysis using parsimony (*and other methods) version 4.0a. Massachusetts: Sinauer Associates; 2003.

[95] Felsenstein Joseph (1985). Confidence Limits on Phylogenies: An Approach Using the Bootstrap. Evolution.

