# Supplementary material for: The phylogenetic significance of the morphology of the syrinx, hyoid and larynx, of the southern cassowary, Casuarius casuarius (Aves, Palaeognathae)
Source: BMC Evol Biol. 2019 Dec 27;19:233. doi: 10.1186/s12862-019-1544-7 (PMC6935130; doi:10.1186/s12862-019-1544-7)
Supplement: Supplementary file 1 — Additional file 1: SI 1. Moa species by taxon with tracheal rings. All listed specimens had tracheal rings preserved; specimens with bronchosyringeal half-rings and syringeal keeled rings are specifically identified. A list of all moa taxa with recovered tracheal rings in the Museum of New Zealand Te Papa Tongarewa. [file 12862_2019_1544_MOESM1_ESM.docx]

**SI 1. Moa species by taxon with tracheal rings. All listed specimens had tracheal rings preserved; specimens with bronchosyringeal half-rings and syringeal keeled rings are specifically identified.**

| **Moa taxon** | **Specimens Observed** | **Ossified tracheal rings** | **Syringeal keeled ring** |
| --- | --- | --- | --- |
| *Dinornis* | S. 034095, S. 035304, S. 041230, S. 041245, | Present in all specimens |  |
| *Megalapteryx didinus* | S. 023346, S. 023429, S. 023430, S. 023575, S. 023647, S. 023733, S. 028206, S. 032808 | Present in all specimens | Present:  S. 028206, S. 023575 |
| *Anomalopteryx didiformis* | S. 000466, S. 024417, S. 024438, S. 034013, S. 034014, S. 035273, S. 036741, S. 038909 S. 042237, S. 044756, | Present in all specimens | Present:  S. 024417 |
| *Euryapteryx* | S. 003902, S. 025656, S. 027794, S. 028438, S. 038884, S. 039021 | Present in all specimens |  |
| *Pachyornis* | S. 027896, S. 036520, S. 041014, | Present in all specimens |  |
| Unidentified moa taxa | S. 036486, S. 036493, S. 036498 S. 036503, S. 036549, S. 036550, S. 036572, S. 038439,  S. 039355, S. 040529, S. 041224, S. 041235,  S. 043801, S. 044754, S. 044755, S. 044762, S. 044767 | Present in all specimens | Present:  S. 44754 |

All specimens from the National Museum of New Zealand (NMNZ)

^*^Presence or absence of keeled syringeal ring is not confirmed.
