# Supplementary material for: The phylogenetic significance of the morphology of the syrinx, hyoid and larynx, of the southern cassowary, Casuarius casuarius (Aves, Palaeognathae)
Source: BMC Evol Biol. 2019 Dec 27;19:233. doi: 10.1186/s12862-019-1544-7 (PMC6935130; doi:10.1186/s12862-019-1544-7)
Supplement: Supplementary file 2 — Additional file 2: SI 2. Tinamou cricoid images from Museo de Historia Natural de La Pampa. Images of the larynx and cricoid of three tinamou species (Nothura maculosa, Eudromia elegans, and Rhynchotus rufescens), taken by Marcos Cenizo, Museo de Historia Natural de La Pampa. [file 12862_2019_1544_MOESM2_ESM.docx]

**SI 2. Tinamou cricoid images from Museo de Historia Natural de La Pampa**


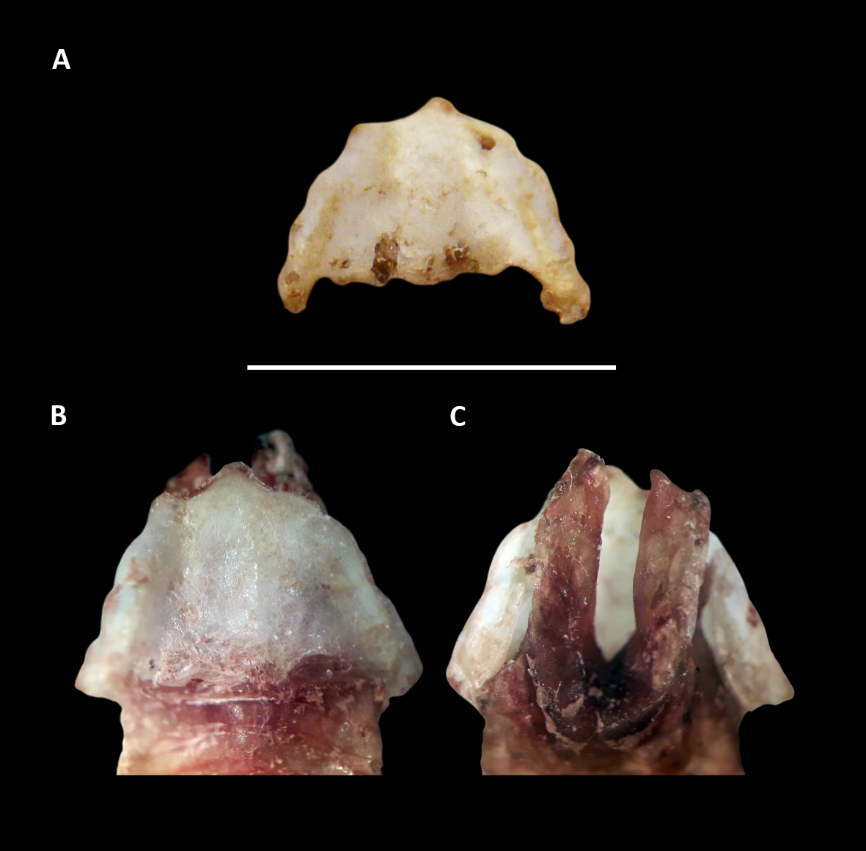


**SI 2, Figure 1:** Tinamou species *Nothura maculosa*, specimen MMC 321. **A.** Cricoid, **B.** Articulated larynx including cricoid, procricoid, arytenoids and tracheal tube, ventral view, **C.** Dorsal view. Scale bar = 5mm.


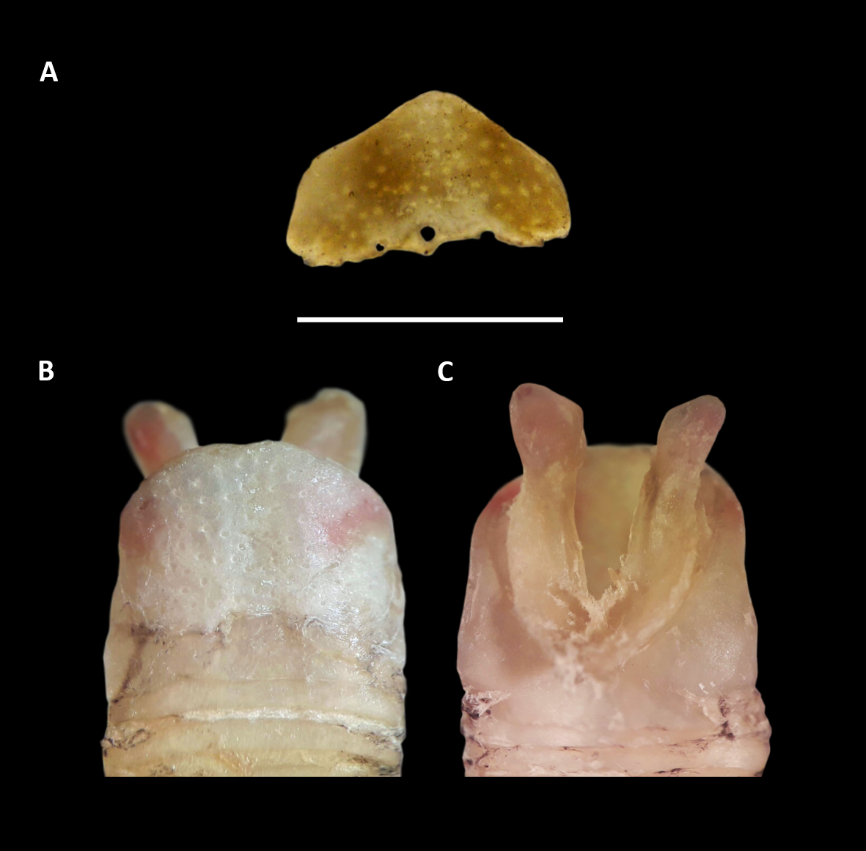


**SI 2, Figure 2:** Tinamou species *Eudromia elegans,* specimen MMC 350. **A.** Cricoid, **B.** Articulated larynx including cricoid, procricoid, arytenoids and tracheal tube, ventral view, **C.** Dorsal view. Scale bar = 5mm.


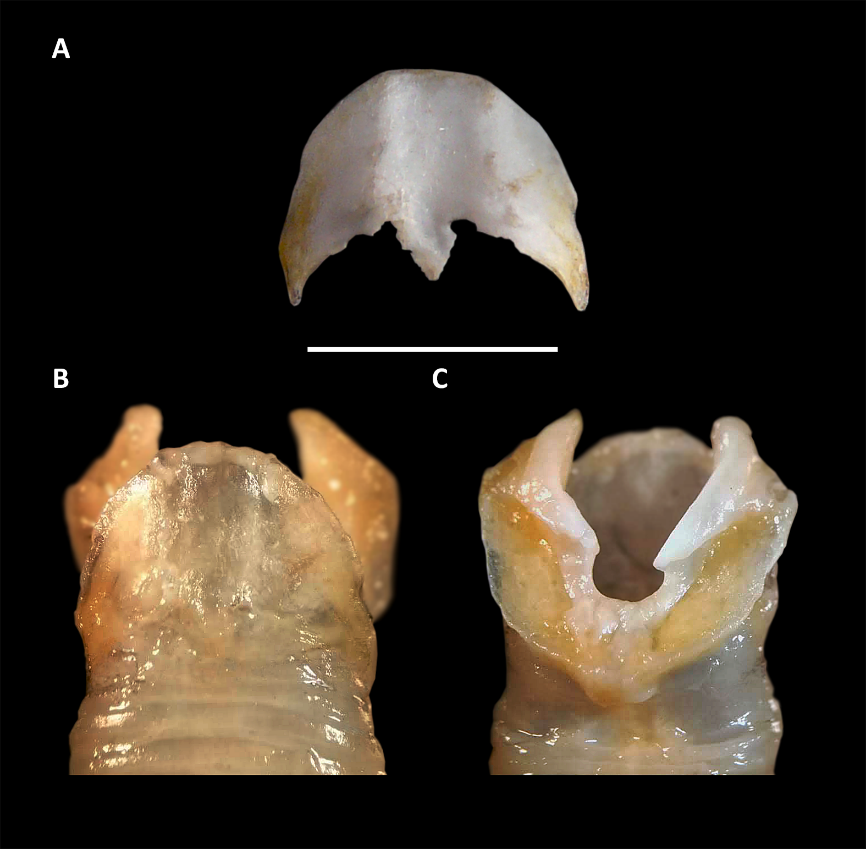


**SI 2, Figure 3:** Tinamou species *Rhynchotus rufescens,* specimen MMC 358. **A.** Cricoid, **B.** Articulated larynx including cricoid, procricoid, arytenoids and tracheal tube, ventral view, **C.** Dorsal view. Scale bar = 5mm.
