# Supplementary material for: The phylogenetic significance of the morphology of the syrinx, hyoid and larynx, of the southern cassowary, Casuarius casuarius (Aves, Palaeognathae)
Source: BMC Evol Biol. 2019 Dec 27;19:233. doi: 10.1186/s12862-019-1544-7 (PMC6935130; doi:10.1186/s12862-019-1544-7)
Supplement: Supplementary file 3 — Additional file 3: SI 3. Syrinx, hyoid, and larynx characters and character states. All characters developed through morphological analysis of the syrinx, hyoid and larynx in palaeognaths, used in the phylogenetic analyses and optimised onto the three resulting topologies. [file 12862_2019_1544_MOESM3_ESM.docx]

**SI 3. Syrinx, hyoid and larynx characters and character states**.

References indicated in square brackets relate to references in main document.

1. Syrinx Type [32: 0738]: Unordered

1. Type bronchial; 1. Type tracheal; 2. Type tracheo-bronchial

2. Ossification of syrinx elements [32: 0735]: Ordered

1. Ossification present; 1. Cartilaginous

3. Cart. bronchosyringeales; number present: Ordered

1. 0; 1. 1-4; 2. 5-8; 3. >8

4. Cart. bronchosyringeales medial ridges bordering internal membranes [90: 40]: Ordered

1. No fusion; 1. First and second bronchial rings fused; 2. Three or more bronchial rings fused

5. Modifications to shape of incomplete cart. bronchosyringeales [90: 34]: Unordered

1. Wider at medial ends; 1. Wider laterally with narrowed ends medially; 2. Uniform width

6. Asymmetry [35: 6]: Ordered

1. Absent; 1. Present, minor; 2. Present, major

Note: Minor asymmetry occurs as a result of slight size or shape variation between the left and right sides of the syrinx. Major asymmetry when one side is considerably larger or of different shape to the other, includes single-sided presence of associated structures.

7. Cart. tracheosyringeales incomplete dorsally: Unordered

1. Absent; 1. Present

8. Cart. tracheosyringeales width relative to cart. bronchosyringeales [35: 4]: Unordered

1. Tracheosyringeal rings thinner in width; 1. Uniform width; 2. Bronchosyringeal rings thinner in width

9. Cart. tracheosyringeales directed downwards along the midline [18]: Unordered

1. One to two rings; 1. Up to 5 rings; 2. None

10. Tympanum [90: 20, 21; 32: 0736]: Unordered

1. Present; 1. Absent

11. Shape of tympanum, or caudal end of trachea if tympanum absent [90: 26]: Ordered

1. Gradually widens caudally; 1. Almost cylindrical; 2. Cylindrical; 3. Mediolateral compression

12. Pessulus [90: 15]: Unordered

1. Absent; 1. Present; 2. Dorsal pessuliform process present

*Note- Absence of the pessulus results in Membrana tympaniformis connecting both bronchial tubes medially at the tracheal bifurcation*

13. Pessulus [32: 0739]: Ordered

1. Cartilaginous; 1. Full or part ossification

14. Lateral intervals between cart. tracheosyringeales and cart. bronchosyringeales [35: 3; 90: 36]: Ordered

1. Wide lateral intervals between tracheal and bronchial rings; 1. Small interval between tracheal and bronchial rings; 2. No interval; overlap, or almost overlap, of tracheal and bronchial rings

15. Dorsal indentation of tracheosyringeal rings: Unordered

1. Present; 1. Absent

16. Musculature [66]: Ordered

1. No intrinsic musculature; 1. 1 or 2 intrinsic muscle pairs; 2. 3 or more intrinsic muscle pairs

17. Basiurohyale [24]: Ordered

1. Cartilaginous; 1. Part ossification; 2. Full ossification

18. Basihyale and urohyale [32: 0758]: Unordered

1. Synostosis, articular surfaces indiscernible; 1. Syndesmosis, articular surfaces discernible

19. Urohyale [32]: Unordered

1. Present; 1. Absent

*Note*- When the urohyal is absent, the basihyal bone terminates just caudal to basihyal-ceratobranchial joint.

20. Urohyale shape [24]: Unordered

1. Element with rounded caudal edge; 1. Element with angular caudal edge; 2. Urohyal absent

21. Basihyale rostral edge [32; 24]: Ordered

1. Basihyoid rostral central concavity; 1. Basihyoid flattened rostral edge; 2. Basihyoid rostral edge convex

22. Ceratobranchiale [24]: Unordered

1. Cartilaginous; 1. Ossified

23. Epibranchiale length relative to ceratobranchial [32: 0763]: Ordered

1. Epibranchials shorter than ceratobranchials; 1. Both comparable in length; 2. Epibranchials considerably longer than ceratobranchials

24. Tongue shape, dorsal view [28]: Unordered

1. Reduced triangular; 1. Reduced ovular; 2. Intermediate (Triangular); 3. Elongated corpus

*Note*- Elongated corpus includes all elongated tongue types as described by Erdogan and Iwasaki [28].

25. Tongue shape, frontal view: Unordered

1. Rounded tongue; 1. Dorso-ventrally flattened

26. Papillae along margins of tongue corpus [28]: Unordered

1. Absent; 1. Posterolateral corner of the tongue terminate with small lateral papillae; 2. Numerous lingual papillae along lateral margins

27. Paraglossum [29]: Unordered

1. Paired paraglossalia; 1. Single paraglossum

28. Paraglossum, when only single paraglossum [24]: Unordered

1. Triangular, angled, deep concavity on caudal margin; 1. Triangular, shallow concavity on caudal margin; 2. Angled triangular, rounded, deep concavity on caudal margin; 3. Elongate triangular

29. Cart. cricoidea [25]: Ordered

1. Cartilaginous or rarely small ossification centres in older individuals; 1. Consistent part or full ossification of corpus; 2. Ossified corpus

30. Cart. cricoidea corpus, crista ventralis [26]: Ordered

1. Absent; 1. Only shallow ridge(s) present; 2. Prominent ridge(s) only part length of corpus; 3. Prominent ridge(s) full length of corpus

31. Cart. cricoidea corpus, proc. rostralis [25]: Ordered

1. Prominent, flattened rostral process; 1. Prominent with angular rim; 2. Reduced, but present; 3. Absent, flattened cranial edge of corpus

32. Ala; cart. cricoidea dorsalis [25]: Unordered

1. Wings fused, partly fused, or articulating with each other, closing cricoid ring dorsally; 1. Wings unfused, cricoid ring closed dorsally by procricoid articulation

33. Cart. cricoidea fusion with cart. tracheales [26]: Unordered

1. One or more tracheal rings fused with caudal end of cricoid; 1. No tracheal rings fused with cricoid

34. Cart. procricoidea [25]: Unordered

1. Ossified; 1. Cartilaginous; 2. Absent

35. Cart. procricoidea corpus shape, dorsal view [25]: Unordered

1. Cuboidal; 1. Triangular; 2. Circular; 3. Diamond; 4. Oval

36. Cart. procricoidea cauda, the ventral, ventro-caudad pointing tail [25]: Unordered

1. Absent; 1. present

37. Cart. arytenoidea ossification [25]: Ordered

1. Ossified; 1. Part ossified; 2. Cartilaginous

38. Cart. arytenoidea, proc. rostralis and caudalis [25]: Ordered

1. Proc. caudalis and rostralis present; 1. Only proc. caudalis present; 2. Neither present

39. Cart. arytenoidea shape [26]: Unordered

1. Rounded; 1. Flattened

40. Cart. arytenoidea and glottis lips [29]: Unordered

1. Arytenoid cartilage supports glottis lips; 1. Arytenoid cartilage a separate structure to glottis lips

41. Laryngeal papillae, extending from elevated glottis lips [29]: Ordered

1. No papillae present; 1. 1-2 papillae; 2. 3-8 papillae; 3. > 8 papillae

42. Laryngeal papillae [58]: Ordered

1. Extend only from lateral margins; 1. Extend from both lateral and caudal margins; 2. Extend only from caudal margins

43. Articulation of the maxillary process of the nasal with the maxilla: Unordered

1. Absent; 1. Present
