# Supplementary material for: The phylogenetic significance of the morphology of the syrinx, hyoid and larynx, of the southern cassowary, Casuarius casuarius (Aves, Palaeognathae)
Source: BMC Evol Biol. 2019 Dec 27;19:233. doi: 10.1186/s12862-019-1544-7 (PMC6935130; doi:10.1186/s12862-019-1544-7)
Supplement: Supplementary file 4 — Additional file 4: SI 4. Character matrix for 28 palaeognath and outgroup taxa, 42 SHL characters and one maxillary character. Character matrix for all 28 palaeognath and outgroup taxa and 42 characters assessed in this study. [file 12862_2019_1544_MOESM4_ESM.docx]

**SI 4. Character matrix for 28 palaeognath and outgroup taxa, 42 SHL characters and one maxillary character**

| **Taxa** | **Characters** |
| --- | --- |
| *Nothura_maculosa*  *Eudromia_elegans*  *Crypturellus*  *Megalapteryx*  *Dinornis_robustus*  *Pachyornis_geranoides*  *Pachyornis_elephantopus*  *Pachyornis_australis*  *Anomalopteryx*  *Emeus_crassus*  *Euryapteryx_curtus*  *Casuarius_bennetti*  *Casuarius_casuarius*  *Dromaius_novaehollandiae*  *Dromaius_baudinianus*  *Apteryx_australis*  *Apteryx_owenii*  *Aepyornis*  *Rhea_americana*  *Rhea_pennata*  *Struthio_camelus*  *Lithornis*  *Grus_rubicunda*  *Anhima_cornuta*  *Anseranas_semipalmata*  *Leipoa_ocellata*  *Gallus_gallus*  *Chauna_torquata* | 211000020120?0101000110???132?1110??011???2  21???00?01?0?0101000210???1?2030103?011???2  211020020120?0101000111???13??????????????2  20?0??1?1?011?1?000021101112212?0???0?1???2  20?0??1?1?011?1?000021101???213?0???0?1???2  20?0??1?1?011?1?000021101???201?0???0?1???2  20?0??1?1?011?1?000021101???201?0???0?1???2  20?0??1?1?011?1?000021101???201?0???0?1???2  20?0??1?1?011?1?000021101???212?0???0?1???2  20?0??1?1?011?1?000021101???222?0???0?1????  20?0??1?1?011?1?000021101???211?0???0?1????  ??????????????????????????????????????????1  21?001101110?1000000110012110021113121110?1  21?0??1??100?1100000110012110010110021110?1  ??????????????????????????????????????????1  212020101100?21110011101?0?203311???????121  212020101100?21010011101?0??03311???????121  21????????????????????????????????????????1  20202002100110112?1211001110003001112100321  ??????????????????????????????????????????1  211020120112?11000010100110?103011111000211  ?1???????????????????1????????????????????2  20?10?0?10?11?1?2101?1??????220?0?????????2  ??????????????????????????????????????????2  20???0?2?0311???????????????220?1?????????2  ????????????????2000210???????????????????2  20?121112001101?200101??????2?1110411010322  20?0?0?2???11?????????????????????????????2 |
